# Innate immune signaling–driven myeloid remodeling in cancer: inflammatory amplification, immune suppression, and therapeutic targeting

**DOI:** 10.3389/fimmu.2026.1941227

**Published:** 2026-09-22

**Authors:** Wenyi Ye, Linhan Zhong, Xiao Wang, Jun Chen

**Affiliations:** 1 Internet Medical Center, The First Affiliated Hospital of Zhejiang Chinese Medical University (Zhejiang Provincial Hospital of Chinese Medicine), Hangzhou, Zhejiang, China; 2 The First Affiliated Hospital of Zhejiang Chinese Medical University (Zhejiang Provincial Hospital of Chinese Medicine), Hangzhou, Zhejiang, China; 3 Department of Geriatrics, The First Affiliated Hospital of Zhejiang Chinese Medical University (Zhejiang Provincial Hospital of Chinese Medicine), Hangzhou, Zhejiang, China

**Keywords:** immunotherapy resistance, innate immunity, myeloid remodeling, myeloid-derived suppressor cells, tumor-associated macrophages

## Abstract

Innate immune signaling is a central regulator of cancer immunity, but its effects are highly context-dependent. Acute and spatially controlled innate immune activation can promote dendritic-cell maturation, antigen presentation, and cytotoxic lymphocyte recruitment. In contrast, chronic, systemic, or therapy-induced inflammatory signaling often redirects the tumor ecosystem toward suppressive myeloid remodeling. This review discusses how innate immune pathways reshape myelopoiesis, macrophage polarization, MDSC activation, tumor-associated neutrophil recruitment, and stromal–myeloid inflammatory niches. We focus on selected signaling axes that represent distinct layers of innate inflammatory regulation, including cytokine-mediated myeloid instruction, inflammasome activation, nucleic-acid sensing, chemokine-driven recruitment, stromal transcriptional integration, and lipid-mediator signaling. These axes include IL-4/IL-4Rα, IL-1α/IL-1β, TGF-β, interferon signaling, NLRP3 inflammasome activation, STING, STAT3, IL-8/CXCR2, CCR2, and CysLTR1. These pathways can expand suppressive myeloid output from the bone marrow, reinforce tumor-associated macrophage states such as TREM2^+^, SPP1^+^, and NLRP3^+^ macrophages, and promote MDSC- and neutrophil-mediated resistance to immune checkpoint blockade. We also summarize tissue, spatial, and peripheral biomarkers that may identify myeloid-dominant immune resistance. Finally, we discuss therapeutic strategies aimed at blocking inflammatory drivers, inhibiting inflammasome-dependent amplification, preventing suppressive myeloid recruitment, and reprogramming established myeloid niches. Rather than cataloguing individual myeloid subsets or isolated inflammatory pathways, this review integrates systemic myelopoiesis, local TAM/MDSC/TAN remodeling, stromal–myeloid spatial niches, and therapy-induced inflammatory feedback into a unified framework for understanding and targeting immune checkpoint blockade resistance.

## Highlights

- Bone marrow is framed as a systemic training site for tumor-promoting myeloid cells.
- Innate signals connect systemic myelopoiesis to local immune-exclusion niches.
- Treatment-induced inflammation creates adaptive myeloid feedback during ICB.

## 1 Introduction

Cancer immunotherapy has traditionally been conceptualized through a T-cell-centered framework, in which durable tumor control depends on antigen presentation, effector T-cell priming, intratumoral T-cell infiltration, and reversal of inhibitory checkpoint signaling (1–4). However, clinical resistance to immune checkpoint blockade cannot be fully explained by defective T-cell recognition or insufficient T-cell activation alone. In many tumors, inflammatory and innate immune signals are abundant, but these signals are progressively redirected toward suppressive myeloid programs rather than productive antitumor immunity. This paradox highlights the dual nature of innate immune activation in cancer. Acute, localized, and appropriately timed innate immune signaling can promote dendritic-cell activation, type I interferon production, antigen presentation, and cytotoxic lymphocyte recruitment (5–8). In contrast, chronic, systemic, spatially dysregulated, or therapy-induced innate immune activation can expand suppressive myeloid populations, reinforce inflammatory feedback loops, and establish tumor microenvironments that exclude or disable cytotoxic T cells and natural killer cells.

This review focuses on innate immune signaling–driven myeloid remodeling, defined here as the process by which inflammatory cues reshape myeloid development, recruitment, differentiation, functional polarization, and spatial organization across systemic and local immune compartments (9–13). This remodeling may begin in the bone marrow through emergency myelopoiesis and continue within tumors, where monocytes, macrophages, myeloid-derived suppressor cells, and neutrophil-lineage cells acquire suppressive programs. These populations can amplify inflammation while limiting antitumor immunity through cytokine secretion, metabolic competition, checkpoint ligand expression, extracellular matrix remodeling, and exclusion of effector lymphocytes from malignant cell nests.

Accordingly, this review discusses selected innate immune and inflammatory signaling axes that converge on cancer-associated myeloid remodeling. In this review, we use the term “innate immune signaling” in an operational rather than strictly canonical sense. Specifically, it refers to signaling programs that are initiated by, amplify, or transmit innate inflammatory cues and that directly regulate myeloid development, recruitment, polarization, suppressive activity, or spatial organization in cancer (14–16). This definition includes canonical innate sensing pathways, such as NLRP3 inflammasome activation and cGAS–STING signaling, but also cytokine, chemokine, lipid-mediator, stromal, and transcriptional programs when they function as proximal regulators of myeloid remodeling (17–19). The pathways emphasized here were prioritized because they meet at least one of the following criteria: direct evidence for regulating bone marrow myelopoiesis or tumor-infiltrating myeloid states; mechanistic involvement in TAM-, MDSC-, or TAN-mediated immune suppression; relevance to immune checkpoint blockade resistance; or emerging biomarker or therapeutic potential (20–22). Therefore, this review does not aim to provide an exhaustive catalogue of all myeloid regulators. Instead, it develops a focused framework linking innate inflammatory interpretation to systemic and local myeloid remodeling, immune resistance, and therapeutic reprogramming in cancer.

This narrative review was based on a structured search of PubMed and Web of Science. Search terms included combinations of “innate immunity”, “myeloid remodeling”, “tumor-associated macrophages”, “myeloid-derived suppressor cells”, “tumor-associated neutrophils”, “myelopoiesis”, “immune checkpoint blockade”, “immunotherapy resistance”, “IL-4”, “IL-1”, “TGF-β”, “interferon”, “NLRP3”, “STING”, “STAT3”, “CXCR2”, “CCR2”, and “CysLTR1”. Additional pathway-specific searches included “TREM2 macrophages”, “SPP1 macrophages”, “efferocytosis”, “stromal–myeloid niche”, “CAF macrophage crosstalk” and “therapy-induced myeloid resistance”. We prioritized English-language original research articles, mechanistic studies, single-cell or spatial profiling studies, translational studies using human tumor samples, and preclinical studies directly linking innate inflammatory signaling to myelopoiesis, TAM/MDSC/TAN remodeling, stromal–myeloid immune exclusion, or immunotherapy resistance (23, 24). Landmark studies were also included when they established key concepts or mechanisms. Reviews, editorials, and commentaries were used only for background context. Studies without direct relevance to cancer-associated myeloid biology, innate inflammatory signaling, or immunotherapy response were excluded. As this was a narrative review rather than a systematic review or meta-analysis, formal risk-of-bias assessment and quantitative synthesis were not performed.

## 2 Defining innate inflammatory signals as upstream drivers of myeloid remodeling

Innate immune signaling provides the first layer of immune interpretation within the tumor microenvironment. Pattern-recognition receptors, inflammatory cytokines, damage-associated molecular patterns, microbial products, therapy-induced cell death, and stromal stress signals can all activate innate immune pathways (25–27). However, the biological outcome of this activation depends on timing, intensity, cellular context, and tissue architecture. In immunologically permissive settings, innate immune activation supports dendritic-cell maturation and cytotoxic lymphocyte recruitment. In immunologically suppressive tumors, the same inflammatory machinery can reinforce myeloid expansion, monocyte and neutrophil recruitment, macrophage polarization, and MDSC activation (28–30). Thus, innate immune signals should not be interpreted simply as “immune-stimulatory” or “immune-suppressive.” Instead, they function as upstream regulators of cellular ecosystem remodeling, and their ultimate effect is determined by how they are processed by myeloid cells, stromal cells, and malignant cells. To avoid an overly broad catalogue of inflammatory mediators, the pathways discussed below are organized according to their functional position within the innate immune–myeloid remodeling cascade. IL-4/IL-4Rα, IL-1, TGF-β, and interferon signaling are discussed as cytokine-based instructive axes that regulate myelopoiesis, macrophage polarization, immune exclusion, or adaptive resistance (29–31). NLRP3 and STING are discussed as canonical innate sensing and inflammatory amplification pathways. IL-8/CXCR2 and CCR2 are included because they mediate compensatory recruitment of neutrophil-lineage cells, monocytes, TAMs, and MDSCs after tumor- or therapy-induced inflammation (28, 32). STAT3 is included as a transcriptional integrator of stromal and myeloid inflammatory programs, whereas CysLTR1 is included as a lipid-mediator pathway with recent evidence linking it to tumor-promoting myelopoiesis and ICB resistance (19, 33). This organization is intended to clarify how heterogeneous inflammatory signals converge on the common biological outcome of suppressive myeloid expansion, recruitment, or reprogramming.

### 2.1 Cytokine-driven inflammatory amplification: IL-4, IL-1, TGF-β, and interferon signaling

Cytokines are often described as downstream inflammatory mediators, but in cancer they also serve as upstream instructors of myeloid cell fate, localization, and suppressive activity. IL-4 provides a clear example of this principle. Although IL-4 is classically associated with type 2 immunity and alternative macrophage activation, recent evidence indicates that IL-4 can act at the level of bone marrow myelopoiesis (34–38). IL-4 signaling through IL-4Rα in early myeloid progenitors can promote the generation of tumor-supportive monocyte-lineage cells, thereby linking systemic type 2 inflammation to the production of immunosuppressive myeloid populations. This finding shifts the interpretation of IL-4 from a local macrophage-polarizing cytokine to a systemic regulator of cancer-associated myeloid output (29).

IL-1 signaling represents another major inflammatory amplification axis. IL-1α and IL-1β can arise from tissue damage, cellular senescence, dying tumor cells, inflammasome activation, and aging-associated hematopoietic changes (39–42). In the context of cancer, IL-1 signaling can promote emergency myelopoiesis and enhance the accumulation of immature or suppressive myeloid cells. A recent lung cancer study showed that hematopoietic aging enhances emergency myelopoiesis and promotes the accumulation of myeloid progenitor-like cells in tumors, with IL-1α acting as a central driver of this enhanced myeloid response (30). This mechanism provides an important explanation for why older hosts may develop a more myeloid-dominant and immunosuppressive tumor microenvironment, even when the malignant cells themselves are not intrinsically more aggressive.

TGF-β adds another layer of complexity because it operates simultaneously on stromal, lymphoid, and myeloid compartments. In colorectal cancer, TGF-β has been shown to build a dual immune barrier by impairing T-cell recruitment while instructing immunosuppressive SPP1^+^ macrophages (31). This finding is important because it demonstrates that immune exclusion is not only a stromal or extracellular matrix phenomenon, nor only a macrophage phenomenon. Instead, TGF-β coordinates both adaptive immune exclusion and innate myeloid suppression. By limiting T-cell access to tumor nests and promoting suppressive macrophage states, TGF-β can convert an inflamed tumor into a spatially segregated and functionally resistant immune ecosystem (43–46).

Interferon signaling also illustrates the context-dependent nature of innate immune activation. Type I and type II interferons are usually considered beneficial for antitumor immunity because they enhance antigen presentation, stimulate chemokine production, and support T-cell priming. However, persistent or treatment-induced interferon signaling can also generate adaptive resistance programs. In advanced renal cell carcinoma, myeloid cells were shown to mediate interferon-driven resistance to immunotherapy, indicating that interferon responses can become suppressive when they are embedded within a myeloid-dominated tumor ecosystem (47). Therefore, interferon signaling should be discussed as a double-edged pathway: it can initiate antitumor immunity, but it can also reinforce myeloid-mediated resistance depending on the cellular compartment in which the signal is sustained.

Together, IL-4, IL-1, TGF-β, and interferon signaling show that cytokine-driven inflammation is not merely a consequence of tumor progression. These pathways actively determine whether innate immune activation produces tumor rejection or myeloid-dominated immune suppression. The central question is therefore not whether inflammation is present, but which cells interpret the inflammatory signal, at what developmental stage, and within which spatial and therapeutic context.

### 2.2 Inflammasome-mediated amplification: NLRP3, IL-1β, and suppressive myeloid states

Inflammasome activation is a major mechanism by which innate immune sensing is converted into inflammatory amplification. Among inflammasomes, NLRP3 is particularly relevant to cancer-associated myeloid remodeling because it integrates diverse danger signals, including cellular stress, apoptotic debris, metabolic disturbance, microbial products, and therapy-induced damage (21, 48–51). Activation of NLRP3 in myeloid cells can lead to caspase-1 activation, IL-1β maturation, IL-18 production, and inflammatory cell-state remodeling. In tumors, this process may promote immune activation under some conditions, but chronic NLRP3 activation often supports tumor growth, myeloid accumulation, and immune suppression. One important mechanism linking tumor cell turnover to myeloid inflammation is efferocytosis. Tumors contain abundant apoptotic debris due to rapid proliferation, hypoxia, nutrient stress, and treatment-induced cell death. Although efferocytosis is normally associated with inflammation resolution, in tumors it can paradoxically activate myeloid NLRP3-dependent inflammasome signaling and IL-1β secretion (52–56). A 2022 study showed that efferocytosis of apoptotic tumor debris drives myeloid NLRP3-dependent inflammasome activation and IL-1β production, thereby promoting tumor growth (57). This finding is conceptually important because it links a seemingly homeostatic clearance process to chronic inflammatory amplification and tumor-promoting myeloid activity.

NLRP3 activation in tumor-associated macrophages may also have biomarker value. In head and neck squamous cell carcinoma, NLRP3 expression in tumor-associated macrophages was associated with poor prognosis and was functionally implicated in tumor growth (58). This supports the idea that NLRP3^+^ macrophages are not merely bystanders within inflamed tumors, but may represent a functionally relevant suppressive and tumor-promoting macrophage state. Such findings justify considering NLRP3, IL-1β, and related inflammasome components as tissue biomarkers for myeloid inflammatory activity. The NLRP3–IL-1β axis is also relevant to MDSC biology. In colorectal cancer, Candida tropicalis was shown to promote tumor progression by activating the NLRP3 inflammasome in MDSCs and enhancing IL-1β-dependent immunosuppressive function (59). This observation links microbial dysbiosis, innate immune sensing, inflammasome activation, and suppressive myeloid function into a single mechanistic axis. It also suggests that the microbial context of a tumor-bearing host may influence cancer progression through myeloid inflammasome programs rather than only through epithelial or metabolic effects.

Therapeutically, these observations provide a rationale for targeting inflammasome activity within suppressive myeloid populations. MDSC-targeting gold nanoparticles were reported to enhance anti-PD-1 tumor immunotherapy by inhibiting NLRP3 inflammasomes, thereby reducing MDSC-mediated immune suppression and improving checkpoint blockade efficacy (60). This strategy is particularly relevant to the theme of this review because it does not simply activate immunity in a general manner; rather, it targets a defined innate inflammatory pathway within a suppressive myeloid compartment. Therefore, NLRP3 inhibition may be most effective when combined with approaches that either block MDSC recruitment or reprogram established suppressive myeloid niches.

Overall, NLRP3 inflammasome signaling represents a key transition point between innate immune sensing and myeloid-dominated tumor promotion. It converts cellular stress, apoptotic debris, microbial signals, and therapy-induced damage into IL-1β-dependent inflammatory amplification. In tumors where this signaling is sustained, NLRP3 activation may strengthen TAM and MDSC suppressive programs, promote resistance to immunotherapy, and provide a tractable target for combination treatment.

### 2.3 Nucleic-acid sensing and context-dependent STING outcomes: immune priming versus myeloid recruitment

Nucleic-acid sensing pathways are central to antitumor immunity because they detect cytosolic DNA or RNA generated by genomic instability, viral infection, mitochondrial stress, micronuclei formation, radiation, chemotherapy, and other forms of tumor cell damage. Among these pathways, cGAS–STING signaling has attracted substantial therapeutic interest because STING activation can induce type I interferon responses, promote dendritic-cell activation, enhance antigen presentation, and stimulate cytotoxic T-cell infiltration. For this reason, STING agonists and STING-activating drug-delivery systems are being explored as strategies to convert immune-cold tumors into inflamed tumors. However, STING activation is not uniformly beneficial (61–65). The downstream inflammatory and chemokine programs induced by STING may recruit both effector immune cells and suppressive myeloid cells. This is particularly important in tumors such as pancreatic ductal adenocarcinoma, where dense stroma, monocyte recruitment, and myeloid suppression are major barriers to immunotherapy. A STING-activating gemcitabine-based nanocarrier was shown to enhance antitumor immune activation, but STING activation also increased CCL2 and CCL7 expression, which promoted CCR2-dependent recruitment of suppressive myeloid cells and contributed to immune resistance (66). This finding provides a clear example of paradoxical myeloid recruitment following innate immune activation.

The divergent outcomes of STING activation can be understood according to several contextual determinants. STING activation is more likely to promote antitumor immunity when it is acute, spatially restricted, and dominated by type I interferon production, dendritic-cell activation, antigen presentation, and CXCL9/CXCL10-mediated effector T-cell recruitment (67–70). In contrast, STING activation may favor suppressive myeloid recruitment when it is sustained, therapy-induced, or embedded within a desmoplastic and myeloid-rich tumor microenvironment, particularly when downstream chemokines such as CCL2, CCL7, CXCL1, CXCL2, or IL-8 dominate over productive antigen-presenting programs (22, 71, 72). The cellular compartment in which STING is activated is also critical: activation in dendritic cells or immunogenic tumor cells may support immune priming, whereas activation in stromal cells, suppressive myeloid cells, or chronically damaged tumor tissue may amplify monocyte, TAM, MDSC, or neutrophil-lineage recruitment (69, 70, 73). Therefore, STING should be interpreted not only as an innate immune agonist pathway but also as a context-dependent inflammatory amplifier whose therapeutic effect depends on timing, duration, cellular localization, tissue architecture, and compensatory chemokine feedback.

The same study addressed this problem by codelivering a CCR2 antagonist with the STING-activating nanocarrier, thereby combining innate immune activation with blockade of myeloid recruitment (66). This therapeutic logic is highly relevant for the broader field: activating innate immunity may be insufficient, or even counterproductive, if the induced chemokine response simultaneously recruits TAMs, MDSCs, or inflammatory monocytes. Therefore, rational STING-based therapy should be designed not only to initiate interferon-driven antitumor immunity, but also to prevent the compensatory accumulation of suppressive myeloid cells. This principle likely extends beyond STING. Many innate immune agonists, including TLR agonists, oncolytic viruses, radiation-induced DNA sensing, and chemotherapy-induced damage responses, may generate both immune-stimulatory and myeloid-recruiting signals. The therapeutic challenge is therefore to separate productive inflammatory priming from suppressive inflammatory feedback. Combination strategies that pair STING activation with CCR2, CSF1R, CXCR2, STAT3, NLRP3, or MDSC-targeting interventions may offer a more effective way to remodel the tumor microenvironment than STING activation alone (74–77).

In this context, STING should be discussed as a context-dependent inflammatory switch rather than a uniformly antitumor pathway. Antitumor STING activity is favored by acute activation, localized type I interferon signaling, effective antigen presentation, dendritic-cell engagement, and effector lymphocyte recruitment (68, 69, 73). By contrast, suppressive STING activity is favored by persistent or therapy-induced activation, dense stromal barriers, pre-existing myeloid dominance, and chemokine programs that recruit CCR2^+^ monocytes, TAMs, MDSCs, or CXCR2^+^ neutrophil-lineage cells (22, 28, 71, 72). These conditions explain why STING activation may convert some immune-cold tumors into inflamed and responsive lesions, but may also reinforce myeloid-dominated resistance in tumors where compensatory chemokine feedback is not controlled. The therapeutic implication is that STING-based strategies should be paired with biomarkers of downstream immune output and, when necessary, with blockade of CCR2-, CXCR2-, STAT3-, or inflammasome-dependent myeloid feedback circuits (22, 28, 71). Together, these selected axes illustrate how distinct categories of innate inflammatory regulation, including cytokine instruction, inflammasome activation, nucleic-acid sensing, chemokine recruitment, transcriptional integration, and lipid-mediator signaling, converge on systemic myelopoiesis, suppressive myeloid recruitment, macrophage polarization, and treatment-induced inflammatory feedback. **Table 1** summarizes the major innate immune and inflammatory signals that drive myeloid remodeling in cancer, together with their cellular targets, functional consequences, and therapeutic implications. **Figure 1** summarizes how innate immune and inflammatory signals reshape myeloid immunity at both systemic and local levels. It highlights the progression from inflammatory sensing and bone marrow myeloid training to TAM/MDSC/TAN-mediated immune suppression, ICB resistance, and myeloid-targeted therapeutic reprogramming.

**Table 1:** Innate immune signals that drive myeloid remodeling in cancer.

Innate immune signal/pathway	Major source or trigger	Main myeloid compartment affected	Mechanistic effect	Cancer context/model	Therapeutic or biomarker implication	Representative PMID
IL-4/IL-4Rα axis	Type 2 inflammatory signals; tumor-associated immune cells	Bone marrow myeloid progenitors; monocyte-derived macrophages	Drives immunosuppressive myelopoiesis and increases tumor-promoting monocyte-lineage output	NSCLC; mouse and human tumor samples	IL-4Rα blockade may reduce suppressive myeloid output and enhance ICB response	(29)
IL-1α-driven emergency myelopoiesis	Hematopoietic aging; inflammatory bone marrow microenvironment	Hematopoietic stem/progenitor cells; myeloid progenitor-like cells	Fuels emergency myelopoiesis and increases tumor-promoting myeloid accumulation	Lung cancer; aging-related tumor progression	IL-1α/IL-1R blockade may be useful in aged or inflammation-high hosts	(30)
Tumor-induced progenitor dysregulation	Tumor-derived systemic inflammatory and oxidative stress signals	Bone marrow myeloid progenitors; circulating monocytes; monocyte-derived TAMs	Establishes progenitor-level programs that fuel immunosuppressive macrophages	Lung cancer; paired mouse and human analysis	Bone marrow and circulating progenitor programs may serve as systemic biomarkers	(14)
CysLTR1 signaling	Cysteinyl leukotrienes; lipid inflammatory signaling	Myeloid progenitors; neutrophil-lineage cells; suppressive myeloid cells	Promotes tumor-supportive myelopoiesis and myeloid-mediated ICB resistance	Multiple tumor models; ICB-resistant tumors	CysLTR1 blockade may reprogram myelopoiesis and restore ICB sensitivity	(19)
TGF-β signaling	CAFs, tumor cells, stromal cells, immune cells	SPP1^+^ TAMs; stromal-associated macrophages	Builds a dual immune barrier by limiting T-cell recruitment and instructing suppressive SPP1^+^ macrophages	Colorectal cancer	TGF-β signatures, SPP1^+^ TAMs, and CD8 exclusion may guide combination therapy	(31)
IFN-related adaptive resistance	ICB-induced inflammation; chronic interferon signaling	Myeloid cells in treatment-resistant tumors	Converts interferon responses into myeloid-mediated resistance under specific contexts	Advanced renal cell carcinoma	IFN signatures should be interpreted by cell type and treatment stage	(47)
NLRP3 inflammasome	Apoptotic debris, microbial products, metabolic stress, therapy-induced damage	TAMs; MDSCs	Activates caspase-1 and IL-1β, reinforcing inflammatory myeloid suppression	Multiple tumor models; HNSCC; CRC	NLRP3/IL-1β may serve as therapeutic targets and myeloid inflammatory biomarkers	(57–59)
STING signaling	Cytosolic DNA, chemotherapy, genomic instability, STING-activating nanocarrier	TAMs; MDSCs; CCR2^+^ monocytes	Induces antitumor IFN signaling but can also induce CCL2/CCL7-mediated suppressive myeloid recruitment	Pancreatic cancer	STING activation may require concurrent CCR2 blockade	(22)
STAT3 signaling	IL-6 family cytokines, oncogenic stress, stromal inflammation	TAMs; MDSCs; CAF-associated inflammatory niches	Maintains stromal inflammation, CAF plasticity, and suppressive myeloid feedback	Pancreatic ductal adenocarcinoma	MEK plus STAT3 inhibition may remodel stromal–myeloid resistance	(33)
IL-8/CXCR2 axis	Tumor cells, CAFs, macrophages, inflammatory stromal cells	TANs; PMN-MDSCs; CXCR2^+^ myeloid cells	Promotes neutrophil-lineage recruitment and immunotherapy resistance	Hepatocellular carcinoma	IL-8/CXCR2 blockade may overcome myeloid-mediated ICB resistance	(28)
CCR2/CCL2–CCL7 axis	STING activation; chemotherapy-induced inflammation; tumor and stromal chemokines	CCR2^+^ monocytes; TAMs; MDSCs	Mediates compensatory myeloid recruitment after innate immune activation	Pancreatic cancer	CCR2 blockade can prevent myeloid feedback during STING-based therapy	(22)
Senescence-associated inflammatory signaling	Aging, chemotherapy, radiotherapy, targeted therapy, cellular stress	TAMs; MDSCs; suppressive stromal–myeloid niches	Induces inflammatory secretory programs that promote immunosuppressive TME and ICB resistance	Multiple tumor models	Senescence and myeloid markers may guide senolytic or senomorphic combinations	(78)

**Figure 1:**
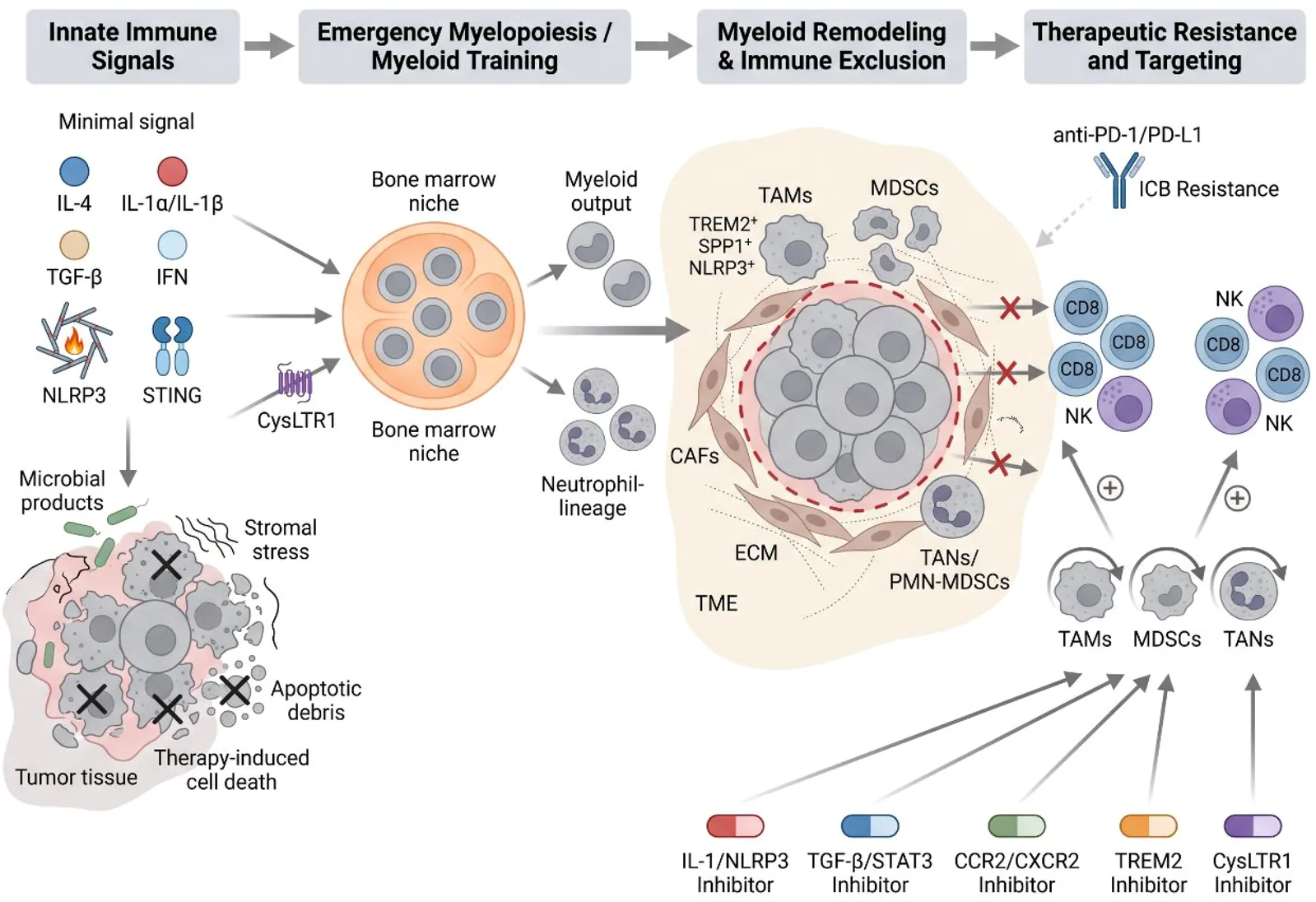
Innate immune signaling–driven myeloid remodeling in cancer. Tumor- and therapy-derived inflammatory cues, including cytokines, inflammasome activation, and STING signaling, reprogram bone marrow myelopoiesis and local tumor-infiltrating myeloid cells. Suppressive TAMs, MDSCs, and TANs/PMN-MDSCs form stromal–myeloid barriers that inhibit CD8^+^ T-cell and NK-cell immunity, promote ICB resistance, and create therapeutic opportunities for myeloid-targeted reprogramming.

Because this review integrates evidence from multiple cancer types, the mechanisms discussed below should not be interpreted as uniformly applicable to all tumors. Some pathways, such as inflammasome-related IL-1 signaling, STING-associated IFN-versus-chemokine output, CCL2/CCR2-mediated monocyte recruitment, IL-8/CXCR2-driven granulocytic recruitment, stromal–myeloid immune exclusion, and treatment-induced inflammatory feedback, are discussed as broader principles because similar inflammatory or myeloid-remodeling patterns have been observed across more than one tumor context (62, 79, 80). In contrast, mechanisms such as IL-4-driven bone marrow myelopoiesis, CysLTR1-dependent myeloid reprogramming, TREM2^+^ TAM-associated lymphocyte suppression, SPP1^+^ CAF-associated macrophage niches, TACE-induced macrophage remodeling, and glioblastoma-associated myeloid programs should be interpreted as context-enriched examples supported by specific tumor models or clinical settings. Therefore, extrapolation across cancer types requires validation using tumor-specific tissue, spatial, and clinical-response data.

## 3 Systemic myelopoiesis: bone marrow as an immune-suppressive training site

Myeloid remodeling in cancer has often been discussed as a local event occurring within the tumor microenvironment (81–85). In this view, monocytes, macrophages, neutrophils, and myeloid-derived suppressor cells are recruited into tumors and then shaped by local cytokines, hypoxia, metabolites, stromal cues, and cell-cell interactions. Although this concept remains important, it is increasingly clear that suppressive myeloid states are not generated exclusively inside tumor lesions. Tumors can impose systemic effects on hematopoiesis, particularly within the bone marrow, thereby altering the production, differentiation trajectory, and functional potential of myeloid cells before they enter the tumor microenvironment (14, 29, 86). This systemic component is crucial because it explains why many patients with advanced cancer show circulating inflammatory myeloid expansion, neutrophilia, monocytosis, immature myeloid-cell accumulation, and poor responses to immune checkpoint blockade even before local tumor-infiltrating immune cells are examined (87–89). The bone marrow can therefore be viewed as an immune-suppressive training site in cancer. Under homeostatic conditions, hematopoietic stem and progenitor cells maintain balanced output of lymphoid and myeloid lineages. During cancer progression, however, tumor-derived cytokines, host inflammatory signals, lipid mediators, aging-related stress, and treatment-induced damage can shift hematopoiesis toward emergency myelopoiesis (18, 29, 30). This process increases the generation of monocytes, neutrophils, and immature myeloid cells that can later differentiate into tumor-associated macrophages, polymorphonuclear MDSCs, monocytic MDSCs, or tumor-associated neutrophils. Importantly, these cells are not merely increased in number; they can also be transcriptionally and epigenetically conditioned to become more resistant to interferon responses, more responsive to tumor-derived inflammatory cues, and more capable of suppressing cytotoxic lymphocytes.

### 3.1 Tumor-derived signals remodel bone marrow myelopoiesis

Tumors can communicate with the bone marrow through soluble cytokines, growth factors, extracellular vesicles, lipid mediators, and systemic inflammatory circuits. These signals alter myeloid output at multiple levels, including hematopoietic stem cells, common myeloid progenitors, granulocyte-monocyte progenitors, and downstream monocyte or neutrophil precursors. The result is a state of tumor-educated myelopoiesis in which the bone marrow preferentially generates myeloid cells that support tumor progression rather than effective immune surveillance. One of the clearest examples of this principle is the IL-4-driven bone marrow axis. IL-4 is traditionally considered a type 2 cytokine involved in allergic inflammation, tissue repair, and alternative macrophage activation (90–93). However, recent work has shown that IL-4 can act much earlier, at the level of bone marrow myeloid progenitors. In cancer, IL-4 signaling through IL-4Rα in early myeloid progenitors promotes immunosuppressive myelopoiesis and increases the generation of tumor-promoting monocyte-lineage cells (29). This finding is important because it reframes IL-4 as a systemic regulator of cancer-associated myeloid development, not merely a local macrophage-polarizing signal within tumor tissue. It also suggests that type 2 inflammatory signals can reshape the immune system before suppressive myeloid cells reach the tumor. This systemic IL-4 axis has therapeutic implications. The same study showed that IL-4Rα blockade could reduce circulating monocytes, increase tumor-infiltrating CD8^+^ T cells, and improve antitumor immune responses when combined with immune checkpoint blockade (29). These observations support the idea that successful immunotherapy may require intervention beyond the primary tumor site. Rather than only reinvigorating exhausted T cells, it may be necessary to interrupt the upstream production of suppressive myeloid cells in the bone marrow. In this sense, the bone marrow becomes a therapeutic compartment, and myelopoiesis itself becomes a targetable immune-regulatory process.

A related concept is that tumors can program suppressive macrophage states at the progenitor level. Recent work has shown that lung tumors can induce myeloid progenitor dysregulation in the bone marrow, priming accessibility for NRF2-associated programs as a cytoprotective response to oxidative stress (14). This altered progenitor state enhances myelopoiesis while dampening interferon responses and promoting the generation of immunosuppressive monocyte-derived macrophages within tumors. The significance of this finding is that tumor-associated macrophage identity may begin to form before monocytes complete their differentiation in the tumor microenvironment. Thus, TAM immunosuppression is not only imposed by local tumor-derived signals, but may also be preconfigured by bone marrow-level transcriptional and chromatin changes. This progenitor-level programming also helps explain why some suppressive myeloid phenotypes are difficult to reverse by targeting local tumor signals alone. If incoming monocytes already carry a tumor-educated transcriptional bias, then local reprogramming strategies may be incomplete unless they also address systemic inflammatory training. In this context, interventions targeting oxidative stress responses, NRF2-associated progenitor programs, or upstream tumor-derived signals may complement TAM-directed therapies (94). Such strategies could reduce the continuous replenishment of suppressive monocyte-derived macrophages and thereby improve the durability of immune checkpoint blockade.

Inflammatory lipid signaling provides another layer of systemic myeloid regulation. Cysteinyl leukotriene receptor 1 has recently been implicated in tumor-promoting myelopoiesis and immune checkpoint therapy resistance (19). Targeting CysLTR1 was reported to reprogram tumor-promoting myeloid output and enhance the response to immune checkpoint blockade, suggesting that leukotriene signaling can function as an upstream regulator of suppressive myeloid generation. This is conceptually important because it broadens the range of innate immune pathways involved in myelopoiesis beyond canonical cytokines such as IL-1, IL-4, IL-6, and GM-CSF. Lipid inflammatory mediators may also shape whether neutrophils and monocytes acquire tumor-promoting or immune-stimulatory properties.

Together, these studies support a model in which tumor-derived signals remodel bone marrow myelopoiesis through cytokine, oxidative stress, and lipid-inflammatory pathways. The tumor does not simply recruit pre-existing myeloid cells; it actively changes the supply chain of myeloid immunity. This systemic remodeling expands the pool of tumor-supportive monocytes, neutrophils, and immature myeloid cells, increases the likelihood that infiltrating cells will adopt suppressive phenotypes, and creates a persistent source of resistance to immunotherapy.

### 3.2 Aging and senescence amplify emergency myelopoiesis

Aging is one of the strongest host-level factors influencing cancer incidence, tumor progression, and response to therapy (95–99). While age-associated cancer risk is often attributed to accumulated mutations or reduced immune surveillance, recent evidence indicates that aging also reshapes hematopoiesis in ways that favor tumor-promoting myeloid inflammation. Hematopoietic aging is associated with myeloid lineage bias, altered stem-cell function, chronic low-grade inflammation, and increased responsiveness to inflammatory cytokines. These changes can amplify emergency myelopoiesis and generate a host environment in which tumors more easily recruit and exploit suppressive myeloid cells. A recent lung cancer study showed that aging of the hematopoietic system can promote cancer progression independently of the age of the tumor or stromal compartment (30). Mechanistically, hematopoietic aging fueled IL-1α-driven emergency myelopoiesis, leading to increased accumulation of myeloid progenitor-like cells in tumors. This work provides a mechanistic bridge between aging-associated inflammation and tumor-promoting myeloid expansion. It suggests that older hosts may be predisposed to a more suppressive tumor immune ecosystem because their bone marrow is more easily shifted into an emergency myelopoietic state. This has important implications for immunotherapy (100–103). If aging increases the baseline production of suppressive myeloid cells, then the same immune checkpoint inhibitor may encounter very different immune ecosystems in younger and older hosts. In one patient, checkpoint blockade may release pre-existing T-cell activity; in another, it may be opposed by a systemic influx of suppressive monocytes, neutrophils, or MDSCs generated through inflammatory myelopoiesis. Therefore, age-associated myeloid bias should not be treated merely as a background variable. It may be a mechanistic determinant of immune resistance and a potential biomarker for selecting patients who require myeloid-targeted combination therapy.

Cellular senescence represents another mechanism that links tissue damage, inflammation, and myeloid suppression. Senescent cells secrete a senescence-associated secretory phenotype that includes cytokines, chemokines, growth factors, matrix-remodeling enzymes, and inflammatory mediators. In cancer, senescence can be induced by aging, oncogenic stress, chemotherapy, radiotherapy, targeted therapy, and immune pressure. Although senescence can suppress tumor-cell proliferation in some contexts, the inflammatory secretome produced by senescent cells can also remodel the tumor microenvironment and promote immune escape. A 2024 study showed that senescence can drive immunotherapy resistance by inducing an immunosuppressive tumor microenvironment (78). Importantly, therapy-induced senescence promoted suppressive myeloid remodeling, while elimination of senescent cells restored immune homeostasis, improved CD8^+^ T-cell proliferation, and enhanced immunotherapy response (104–106). This finding is highly relevant to the concept of systemic and local myeloid training because it shows that treatment itself can generate inflammatory conditions that favor suppressive myeloid accumulation. Thus, anticancer therapy may simultaneously kill tumor cells and create a senescence-driven inflammatory niche that limits subsequent immune control.

Aging and therapy-induced senescence therefore converge on a common immunological outcome: they increase the likelihood that inflammatory signals will be converted into myeloid-dominated suppression. In older patients or in tumors exposed to repeated cytotoxic therapy, emergency myelopoiesis and senescence-associated inflammation may cooperate to expand TAMs, MDSCs, and TANs. These cells can then inhibit effector T-cell function, suppress NK-cell activity, promote angiogenesis, remodel extracellular matrix, and reduce sensitivity to immune checkpoint blockade (107–110). Thus, myeloid remodeling should not be viewed as a purely local consequence of tumor inflammation, but as a systemic immune-training process initiated in the bone marrow and reinforced within the tumor microenvironment. This perspective provides a stronger biological rationale for combining immune checkpoint blockade with strategies that target myelopoiesis, inflammatory cytokines, senescent cells, or suppressive myeloid-cell recruitment. **Figure 2** illustrates how tumor-derived systemic signals, aging-associated inflammation, and therapy-induced senescence remodel bone marrow myelopoiesis before myeloid cells enter the tumor microenvironment. It highlights the bone marrow as an immune-suppressive training site that continuously supplies TAMs, MDSCs, and TANs/PMN-MDSCs to promote immune resistance.

**Figure 2:**
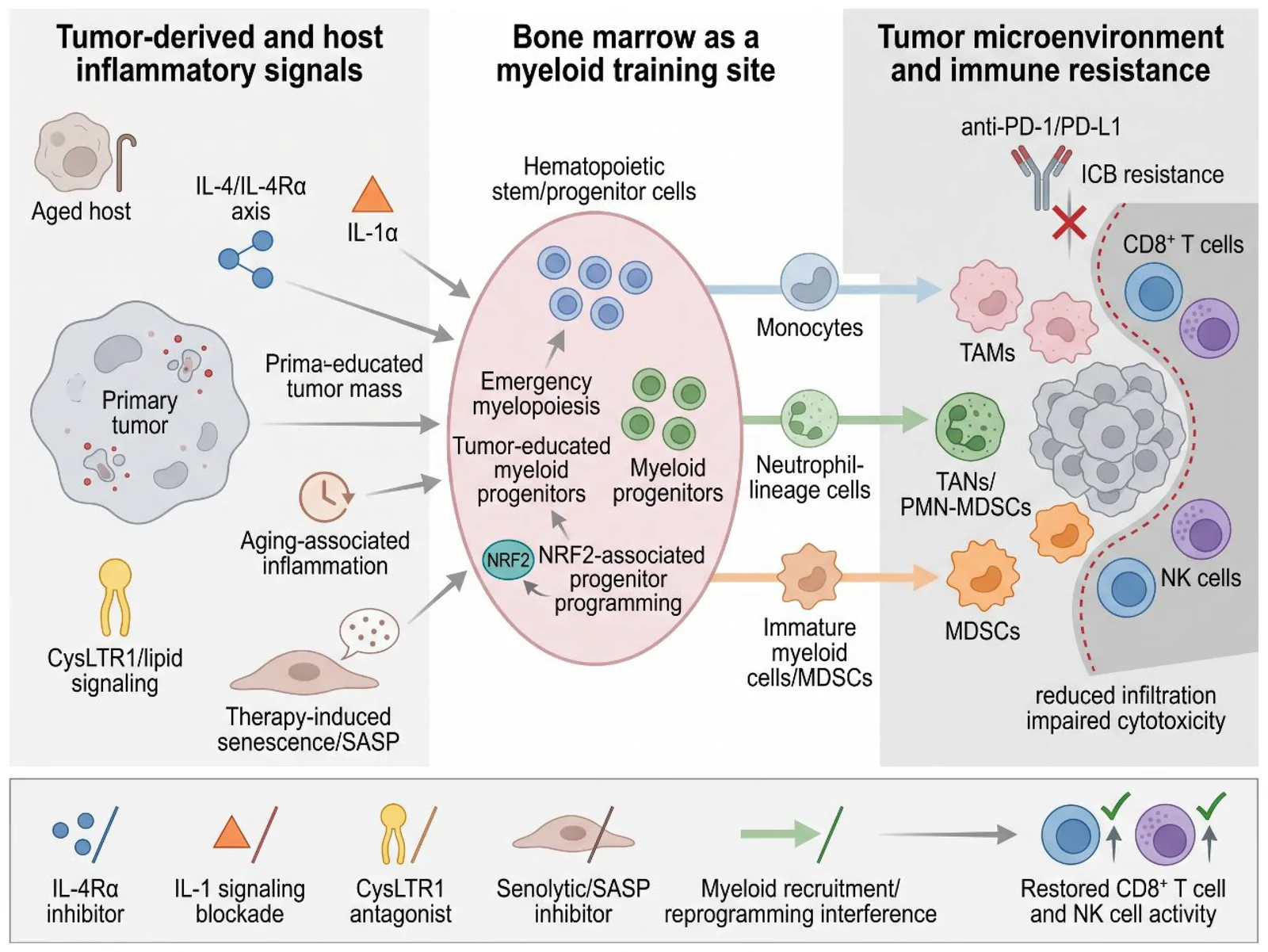
Bone marrow myeloid training in cancer. Tumor-derived cytokines, lipid inflammatory signals, aging-associated inflammation, and therapy-induced senescence drive emergency myelopoiesis and tumor-educated myeloid progenitor programming. The resulting monocytes, neutrophil-lineage cells, and MDSCs infiltrate tumors and differentiate into suppressive TAMs, MDSCs, and TANs/PMN-MDSCs, thereby impairing CD8^+^ T-cell/NK-cell activity and promoting ICB resistance.

## 4 Macrophage remodeling: TAM states shaped by innate inflammatory circuits

Tumor-associated macrophages are among the most plastic and functionally diverse immune cells in cancer. Historically, TAMs were often categorized using the M1/M2 framework, with M1-like macrophages considered pro-inflammatory and antitumor, and M2-like macrophages considered anti-inflammatory and tumor-promoting. Although this framework remains useful as a simplified conceptual model, it is insufficient to capture the diversity of macrophage states now revealed by single-cell transcriptomics, spatial profiling, functional perturbation, and lineage-tracing studies. In tumors, macrophages do not exist as two fixed states. Instead, they occupy multiple context-dependent programs shaped by ontogeny, tissue origin, tumor debris, cytokines, hypoxia, metabolic stress, stromal interactions, and therapy-induced inflammation (111–115). A more useful framework is to define TAM states by their dominant functional circuits. TREM2^+^ macrophages are associated with lipid handling, tumor debris sensing, and suppression of NK- or T-cell immunity. SPP1^+^ macrophages are closely linked to stromal remodeling, immune exclusion, adenosine signaling, and immune checkpoint blockade resistance. NLRP3^+^ macrophages convert apoptotic debris and danger signals into inflammasome-dependent IL-1β production. CAF-associated macrophage states emerge through reciprocal communication with fibroblasts and extracellular matrix components, forming spatially organized suppressive niches (116–119). These macrophage programs are not mutually exclusive, and individual tumors may contain overlapping or transitional TAM states. Nevertheless, organizing TAM biology around these circuits provides a more mechanistic and clinically useful framework than the conventional M1/M2 dichotomy.

### 4.1 TREM2^+^ macrophages: suppression of T-cell and NK-cell immunity

TREM2 is a lipid-sensing receptor expressed by myeloid cells and has emerged as an important regulator of suppressive macrophage states in cancer. TREM2^+^ macrophages are often enriched in lipid-rich, debris-rich, or chronically inflamed tumor microenvironments (120–124). They can acquire programs associated with phagocytosis, lipid metabolism, tissue repair, and immune suppression. Rather than functioning simply as scavenger cells, TREM2^+^ TAMs can reshape the lymphoid compartment and reduce the effectiveness of antitumor immunity. In lung cancer, TREM2^+^ monocyte-derived macrophages were shown to drive NK-cell paucity and dysfunction (125). This study is particularly important because it expands the immunosuppressive role of TAMs beyond T-cell inhibition. NK cells are major innate cytotoxic lymphocytes and can contribute to tumor immune surveillance, especially in tumors with low antigen presentation or impaired MHC class I expression (126–130). The finding that TREM2^+^ macrophages can reduce NK-cell accumulation and cytolytic function indicates that macrophage remodeling can compromise both innate and adaptive cytotoxic immunity. Mechanistically, this work linked TREM2^+^ macrophages to altered IL-18/IL-18BP decoy interactions and IL-15 availability, suggesting that TAMs can suppress NK cells by disrupting cytokine circuits required for NK-cell maintenance and activation. TREM2 targeting has also shown therapeutic relevance in glioblastoma. In orthotopic glioblastoma models, TREM2 inhibition or loss of function remodeled myeloid cells toward a more pro-inflammatory and antitumor state, reduced tumor growth, and improved survival (131). This is important because glioblastoma is typically characterized by strong myeloid dominance, limited T-cell infiltration, and poor response to immune checkpoint blockade. The ability of TREM2 inhibition to reprogram glioblastoma-associated myeloid cells suggests that TREM2 functions not only as a marker of suppressive TAMs, but also as a functional regulator of the macrophage state.

TREM2^+^ macrophages may also mediate therapy-associated immune escape. In hepatocellular carcinoma, TREM2^+^ TAMs were shown to suppress CD8^+^ T-cell infiltration after transarterial chemoembolization, a commonly used locoregional therapy (132). TREM2 deficiency enhanced the therapeutic effect of anti-PD-L1 blockade by improving CD8^+^ T-cell antitumor activity. This finding illustrates a broader principle: local tumor-damaging therapies can generate inflammatory and debris-rich environments that promote suppressive macrophage states. If these states are dominated by TREM2^+^ TAMs, then combining locoregional therapy with checkpoint blockade may be insufficient unless TREM2-dependent macrophage suppression is also addressed. Collectively, these studies identify TREM2^+^ macrophages as a suppressive TAM state with broad relevance across lung cancer, glioblastoma, and hepatocellular carcinoma. Their effects extend to both NK-cell and CD8^+^ T-cell compartments, making them central regulators of innate-adaptive immune crosstalk. Therapeutically, TREM2 blockade may be most effective in tumors where macrophage accumulation, lipid-rich debris, and lymphocyte dysfunction coexist.

### 4.2 SPP1^+^ macrophages: stromal interaction, immune exclusion, and ICB resistance

SPP1^+^ macrophages have emerged as another major suppressive TAM state in cancer. SPP1 encodes osteopontin, a secreted phosphoprotein involved in cell adhesion, matrix remodeling, migration, angiogenesis, and immune regulation. In tumors, SPP1^+^ TAMs are frequently associated with stromal activation, fibrosis, extracellular matrix organization, T-cell exclusion, and resistance to immune checkpoint blockade (133–137). Unlike macrophage states defined only by inflammatory cytokines, SPP1^+^ TAMs often appear as part of multicellular stromal-immune niches involving cancer-associated fibroblasts, endothelial cells, extracellular matrix proteins, and exhausted T cells. In prostate cancer, single-cell and multi-omic analyses identified an SPP1^hi TAM population that increased during disease progression and was linked to myeloid-mediated immunotherapy resistance (138). Functionally, these macrophages were associated with T-cell exhaustion and adenosine signaling, and they contributed to resistance to immune checkpoint blockade. This study is important because metastatic castration-resistant prostate cancer is generally refractory to immune checkpoint inhibitors. The identification of SPP1^hi TAMs provides a mechanistic explanation for this resistance and suggests that myeloid-derived adenosine signaling may be a targetable barrier to immunotherapy.

SPP1^+^ macrophages are also central to stromal-myeloid crosstalk. In gastric cancer peritoneal metastases, CAF-macrophage interactions were shown to govern responses to immune checkpoint blockade (139). This study highlighted the importance of spatially organized communication between fibroblasts and macrophages in shaping therapeutic outcomes. Rather than acting independently, CAFs and TAMs can form reciprocal inflammatory circuits that reinforce immune exclusion and suppressive myeloid differentiation (140–144). This is especially relevant in peritoneal metastasis, where fibrotic stroma, ascites-associated inflammation, and macrophage-rich niches create a highly suppressive immune ecosystem. TGF-β signaling provides a mechanistic bridge between stromal exclusion and SPP1^+^ macrophage instruction. In colorectal cancer, TGF-β was shown to build a dual immune barrier by impairing T-cell recruitment and instructing immunosuppressive SPP1^+^ macrophages (31). This dual effect is central to understanding inflammation-driven myeloid remodeling. TGF-β does not merely inhibit T-cell activity directly; it also alters the cellular architecture of the tumor microenvironment by shaping macrophage states that reinforce immune exclusion. Therefore, TGF-β-driven resistance should be understood as a multicellular process involving stromal remodeling, impaired lymphocyte trafficking, and suppressive TAM polarization.

These findings position SPP1^+^ TAMs as a key interface between myeloid inflammation and stromal immune exclusion. They also suggest that effective therapy may require simultaneous targeting of macrophage-derived suppressive signals, fibroblast activation, extracellular matrix barriers, and adenosine-mediated immune inhibition. In this setting, biomarkers such as SPP1, CAF signatures, TGF-β response programs, adenosine pathway genes, and spatial proximity between TAMs and fibroblasts may help identify patients who are unlikely to respond to immune checkpoint blockade alone.

### 4.3 Efferocytosis and NLRP3^+^ macrophages

Tumor-associated macrophages are continuously exposed to dying tumor cells, apoptotic debris, necrotic material, damaged mitochondria, extracellular nucleic acids, and oxidized lipids. The clearance of apoptotic cells, known as efferocytosis, is generally considered a homeostatic process that prevents uncontrolled inflammation and promotes tissue repair. In cancer, however, efferocytosis can have the opposite effect (145–148). By ingesting tumor apoptotic debris, macrophages may acquire inflammatory and immunosuppressive programs that support tumor growth rather than restore immune homeostasis. A key study showed that efferocytosis of tumor apoptotic debris can activate myeloid NLRP3-dependent inflammasome signaling and IL-1β production, thereby promoting tumor progression (57). This finding is conceptually important because it links normal tissue-clearance machinery to chronic tumor-promoting inflammation. It suggests that the high rate of tumor-cell death within cancers does not automatically favor antigen release and immune activation. Instead, when apoptotic debris is processed by suppressive or tumor-educated macrophages, it may fuel inflammasome activation, IL-1β secretion, and inflammatory amplification.

NLRP3^+^ TAMs may also serve as clinically relevant biomarkers. In head and neck squamous cell carcinoma, NLRP3 was found to be highly expressed in tumor-associated macrophages in both mouse and human tumors, and its expression was associated with poor prognosis and tumor growth (58). This supports the view that NLRP3 activation in macrophages is not merely a transient inflammatory event, but can define a pathogenic TAM state. In such tumors, NLRP3^+^ macrophages may contribute to immune suppression by sustaining IL-1β-driven inflammation, recruiting additional myeloid cells, enhancing tumor-cell survival, and limiting effective cytotoxic lymphocyte responses. The efferocytosis–NLRP3 axis also helps explain why some therapies that increase tumor-cell death do not necessarily improve antitumor immunity (149–153). Chemotherapy, radiotherapy, embolization, targeted therapy, and immunotherapy can all increase apoptotic or necrotic tumor debris. If this debris is cleared by macrophages in a manner that activates NLRP3 and IL-1β, therapy-induced cell death may paradoxically reinforce suppressive myeloid inflammation. This does not mean that tumor-cell killing is undesirable, but it suggests that the immune consequences of cell death depend on how dying cells are sensed and processed by macrophages. Therapeutically, this framework supports combining tumor-killing strategies with interventions that block inflammasome activation, IL-1β signaling, or suppressive macrophage recruitment. In tumors with high NLRP3^+^ TAM signatures, inflammasome inhibition may reduce inflammatory amplification and improve the efficacy of immune checkpoint blockade (51, 154–157). More broadly, the NLRP3^+^ TAM state represents an important example of how innate immune sensing can be converted into chronic myeloid suppression rather than immune-mediated tumor clearance.

Together, TREM2^+^, SPP1^+^, and NLRP3^+^ macrophage states illustrate the diversity of TAM remodeling driven by innate inflammatory circuits. TREM2^+^ TAMs suppress NK and T-cell immunity, SPP1^+^ TAMs interact with stromal cells to promote immune exclusion and ICB resistance, and NLRP3^+^ TAMs convert apoptotic debris into IL-1β-dependent inflammatory amplification. These macrophage states should be considered dynamic, overlapping, and therapeutically targetable components of the broader myeloid remodeling network in cancer. **Figure 3** illustrates how innate inflammatory cues reshape tumor-associated macrophages into distinct suppressive states rather than a simple M1/M2 dichotomy. It highlights TREM2^+^, SPP1^+^, and NLRP3^+^ TAM programs as key mediators of lymphocyte suppression, stromal immune exclusion, inflammatory amplification, and ICB resistance.

**Figure 3:**
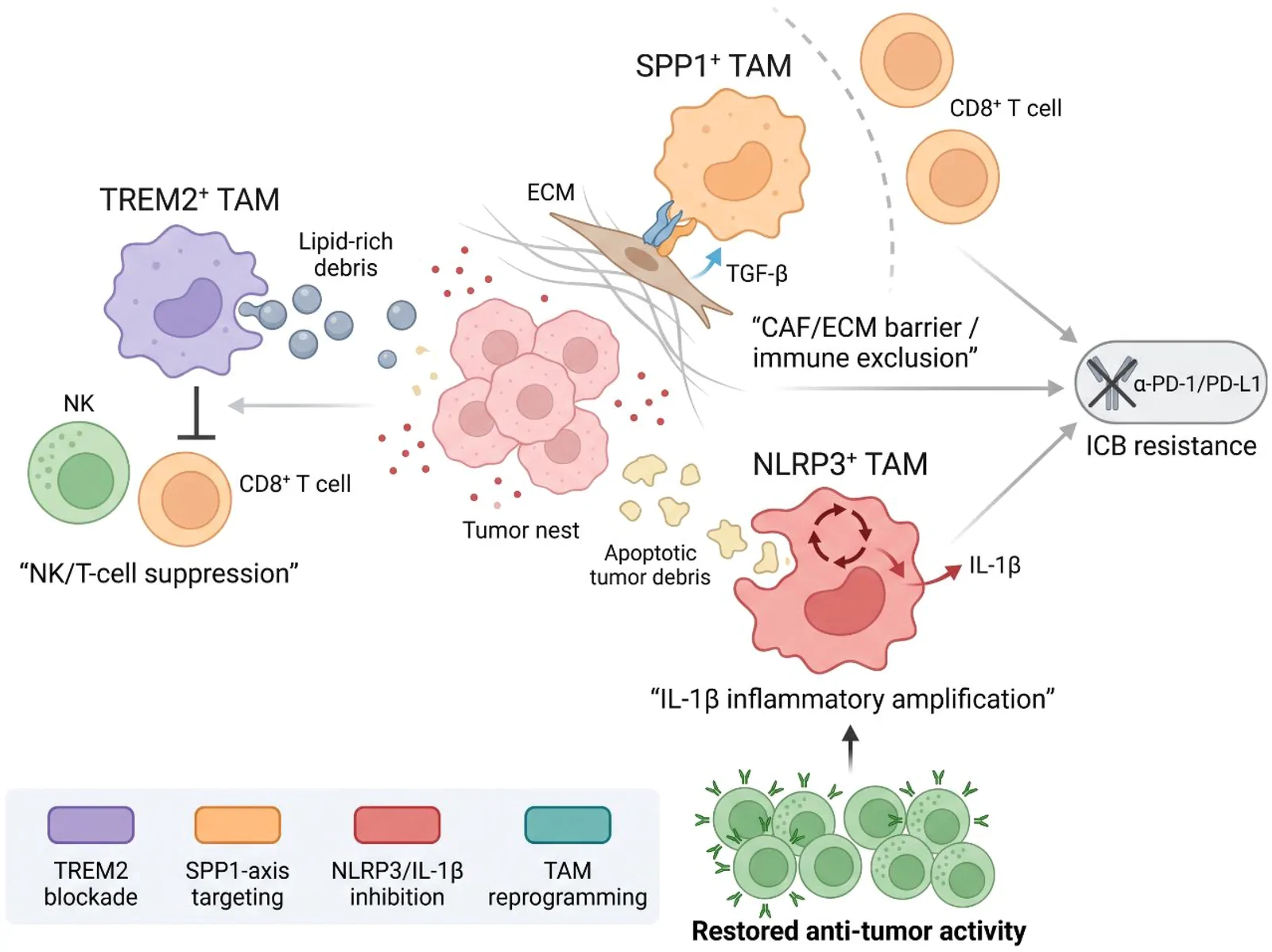
Functional remodeling of tumor-associated macrophages by innate inflammatory circuits. Tumor debris, lipid stress, CAF/TGF-β signaling, and efferocytosis drive TREM2^+^, SPP1^+^, and NLRP3^+^ TAM states. These macrophage programs suppress NK/CD8^+^ T-cell immunity, promote CAF/ECM-mediated immune exclusion, amplify IL-1β-dependent inflammation, and contribute to ICB resistance, providing therapeutic opportunities for TAM reprogramming.

## 5 MDSCs and neutrophils: inflammatory amplification into suppressive granulocytic programs

Beyond macrophages, inflammatory amplification also generates suppressive granulocytic and immature myeloid programs, including myeloid-derived suppressor cells and tumor-associated neutrophils (158–160). These cell populations are highly plastic and are shaped by cytokines, inflammasome activation, microbial signals, lipid mediators, chemokines, hypoxia, and therapy-induced tissue damage. Although MDSCs and TANs are often described as downstream products of tumor inflammation, they should also be viewed as active amplifiers of inflammatory immune suppression. Once recruited or generated, they produce cytokines, chemokines, reactive oxygen species, arginase, prostaglandins, neutrophil extracellular traps, and checkpoint-regulatory molecules that further remodel the tumor microenvironment. MDSCs and TANs are especially relevant to immune checkpoint blockade resistance because they suppress antitumor immunity through mechanisms that are not fully reversed by PD-1 or PD-L1 inhibition. They can reduce T-cell receptor signaling, impair CD8^+^ T-cell proliferation, inhibit NK-cell cytotoxicity, restrict dendritic-cell function, and promote stromal remodeling (158, 161–163). In this sense, suppressive granulocytic programs represent a parallel layer of immune resistance: even when checkpoint blockade reinvigorates exhausted T cells, these cells may remain metabolically, spatially, or functionally constrained by MDSC- and TAN-dominated niches.

### 5.1 NLRP3–IL-1β axis in MDSC immunosuppression

The NLRP3–IL-1β axis is a major mechanism by which innate immune sensing is converted into MDSC-mediated immune suppression. MDSCs are not merely passive recipients of inflammatory cytokines; they can function as inflammasome-competent cells that sense microbial products, dying-cell debris, metabolic stress, and tumor-derived danger signals (164–168). Activation of NLRP3 in MDSCs promotes IL-1β production and strengthens their suppressive activity. This creates a feed-forward loop in which inflammation expands MDSCs, MDSCs produce additional inflammatory mediators, and the resulting microenvironment becomes increasingly resistant to cytotoxic lymphocyte-mediated tumor control. A representative therapeutic study showed that MDSC-targeting gold nanoparticles can enhance anti-PD-1 tumor immunotherapy by inhibiting NLRP3 inflammasomes (60). This work is important because it connects three key concepts: selective delivery to suppressive myeloid cells, inhibition of an innate inflammatory pathway, and enhancement of immune checkpoint blockade. The study suggests that NLRP3 activity within MDSCs is not only a marker of inflammation but also a functional driver of resistance. By suppressing NLRP3 inflammasome activation in MDSCs, gold nanoparticles reduced myeloid-mediated immune suppression and improved the antitumor effect of PD-1 blockade. This provides a strong rationale for designing myeloid-targeted nanomedicine strategies that do not broadly suppress immunity, but instead disable specific inflammatory programs within suppressive myeloid compartments.

The microbiome can also regulate MDSC-mediated inflammation through the NLRP3–IL-1β pathway. In colorectal cancer, Candida tropicalis was shown to promote tumor progression by enhancing the immunosuppressive function of MDSCs through IL-1β-dependent mechanisms (59). This study is notable because it links fungal dysbiosis, inflammasome activation, IL-1β production, and suppressive myeloid function in a single mechanistic axis. It also expands the concept of innate immune signaling beyond tumor-intrinsic or host-derived sterile inflammation. Microbial products and fungal colonization may act as upstream inflammatory stimuli that strengthen MDSC activity and thereby accelerate tumor progression. These findings support the idea that MDSCs are both products and propagators of inflammation. Tumor-derived cytokines, microbial signals, and tissue stress can recruit or expand MDSCs, but once present, these cells reinforce immune suppression by producing inflammatory mediators and suppressive metabolites (155, 169–171). This dual function helps explain why MDSC-rich tumors often show poor responses to immune checkpoint blockade. In such tumors, reinvigorating exhausted T cells may be insufficient unless the NLRP3–IL-1β-dependent suppressive myeloid network is simultaneously interrupted. Therapeutically, targeting the NLRP3–IL-1β axis in MDSCs may be most effective in tumors characterized by chronic inflammation, microbial dysbiosis, high MDSC infiltration, or poor response to PD-1/PD-L1 blockade. Potential strategies include NLRP3 inhibitors, IL-1β blockade, MDSC-targeted nanoparticles, microbiome modulation, and combination therapy with immune checkpoint inhibitors. The key challenge is to suppress tumor-promoting inflammasome activity without eliminating beneficial innate immune activation required for dendritic-cell priming and antitumor immunity.

### 5.2 IL-8/CXCR2 and tumor-associated neutrophils

The IL-8/CXCR2 axis is one of the most important chemokine pathways regulating neutrophil and PMN-MDSC recruitment in cancer (32). IL-8 can be produced by tumor cells, stromal cells, endothelial cells, macrophages, and other inflammatory cells in response to hypoxia, oncogenic signaling, tissue damage, microbial stimulation, and therapy-induced stress (172–175). Through CXCR1 and CXCR2, IL-8 promotes the recruitment, survival, and activation of neutrophil-lineage cells. In tumors, this pathway can increase the accumulation of TANs and PMN-MDSCs, which suppress cytotoxic lymphocytes and promote resistance to immunotherapy. In hepatocellular carcinoma, targeting myeloid IL-8/CXCR2 signaling was shown to overcome immunotherapy resistance (28). This study provides a strong mechanistic example of how chemokine-driven myeloid recruitment can determine response to immune checkpoint blockade. IL-8/CXCR2 signaling promotes the accumulation and suppressive activity of myeloid cells within HCC, thereby limiting the efficacy of immunotherapy. Blocking this pathway can reduce myeloid suppression and improve antitumor immune responses. This makes IL-8/CXCR2 an attractive therapeutic target, particularly in tumors with high neutrophil infiltration, elevated serum IL-8, or enrichment of PMN-MDSC-related signatures (176–180). Tumor-associated neutrophils further illustrate the functional diversity of granulocytic myeloid cells. TANs can display antitumor or pro-tumor activities depending on cancer type, disease stage, inflammatory context, and metabolic environment. In HCC, TANs were shown to attenuate tumor immunosensitivity and curtail immunotherapy response (181). This finding is important because it emphasizes that neutrophils are not simply nonspecific inflammatory bystanders. Instead, distinct TAN subsets can actively shape whether a tumor remains sensitive or resistant to immune-mediated control. In metabolically stressed or inflammation-rich liver cancer microenvironments, TANs may contribute to immune suppression, tissue remodeling, and reduced response to checkpoint blockade.

CysLTR1 signaling provides an additional lipid-inflammatory pathway linking tumor inflammation to suppressive granulocytic remodeling. Targeting cysteinyl leukotriene receptor 1 was reported to reprogram tumor-promoting myelopoiesis and overcome immune checkpoint therapy resistance (19). This finding broadens the field beyond canonical cytokines and chemokines by showing that leukotriene signaling can regulate tumor-promoting myeloid output. Because CysLTR1 is closely connected to inflammatory lipid signaling, this pathway may help explain how tumors convert systemic inflammation into suppressive neutrophil or PMN-MDSC programs (182, 183). It also suggests that drugs targeting lipid-inflammatory receptors may be repurposed or optimized to improve cancer immunotherapy. Together, IL-8/CXCR2 and CysLTR1 pathways demonstrate that suppressive granulocytic programs are generated through coordinated chemokine and lipid-inflammatory circuits. These pathways regulate not only the number of neutrophils and PMN-MDSCs entering tumors, but also their functional state. As a result, targeting granulocytic recruitment or activation may improve immunotherapy outcomes in tumors where neutrophil-lineage cells dominate the immune landscape. Future studies should distinguish TAN subsets from PMN-MDSCs more carefully, define their spatial relationships with CD8^+^ T cells and tumor cells, and identify biomarkers that predict which patients are most likely to benefit from CXCR2, CysLTR1, or related myeloid-targeted interventions (184–188). The suppressive myeloid compartment is composed of heterogeneous macrophage, MDSC, and neutrophil-lineage states rather than a single uniform population. **Table 2** summarizes key suppressive myeloid subsets, representative biomarkers, upstream regulatory signals, functional programs, and potential therapeutic relevance. **Figure 4** illustrates how inflammatory amplification converts MDSCs and TANs/PMN-MDSCs into suppressive granulocytic programs within the tumor microenvironment. It highlights the NLRP3–IL-1β, IL-8/CXCR2, and CysLTR1 axes as key drivers of myeloid-mediated CD8^+^ T-cell, NK-cell, and dendritic-cell dysfunction.

**Table 2:** Suppressive myeloid subsets, biomarkers, and functional programs.

Myeloid subset/state	Representative markers	Dominant upstream signals	Major suppressive functions	Spatial or clinical feature	Potential therapeutic implication	Representative PMID
TREM2^+^ TAMs	TREM2, APOE, LPL, lipid-handling genes, macrophage markers	Lipid-rich tumor debris, therapy-induced damage, chronic inflammation	Suppress NK-cell accumulation and function; limit CD8^+^ T-cell infiltration; promote macrophage-mediated immune resistance	Enriched in lung cancer, GBM, and post-TACE HCC immune niches	TREM2 blockade may reprogram TAMs and enhance NK/T-cell antitumor immunity	(125, 131, 132)
SPP1^+^ TAMs	SPP1, CD68, CD163, C1QC, C3AR1, adenosine-related genes	TGF-β, CAF interaction, stromal remodeling, tumor progression	Promote immune exclusion, T-cell exhaustion, adenosine signaling, stromal remodeling, ICB resistance	Often located in CAF-rich or immune-excluded tumor regions	Target SPP1-associated TAM programs together with stromal/TGF-β or adenosine pathways	(31, 138, 139)
NLRP3^+^ TAMs	NLRP3, IL1B, CASP1, ASC/PYCARD, CD68	Efferocytosis, apoptotic debris, DAMPs, therapy-induced cell death	Convert tumor-cell debris into IL-1β-dependent inflammatory amplification; promote tumor growth	Associated with poor prognosis in HNSCC; relevant in debris-rich tumors	NLRP3 or IL-1β blockade may reduce macrophage-driven inflammatory suppression	(57, 58)
CAF-associated TAMs	SPP1, C1QC, C3AR1, complement genes, macrophage markers; often adjacent to CAF markers such as FAP, ACTA2, COL1A1	CAF-derived TGF-β, chemokines, ECM remodeling, stromal inflammation	Form stable stromal–myeloid suppressive niches; restrict CD8^+^ T-cell access to tumor nests	CAF–TAM proximity and CD8^+^ T-cell exclusion predict poor ICB response	Combine ICB with stromal remodeling, TGF-β blockade, or TAM reprogramming	(31, 139)
Monocytic MDSCs	CD11b, CD14, CCR2, ARG1, IL1B, S100A8/A9, suppressive cytokines	IL-1, CCL2/CCR2, STING-induced CCL2/CCL7, tumor inflammation	Suppress T-cell proliferation; differentiate into suppressive TAMs; amplify inflammatory feedback	May expand after STING activation or chemotherapy-induced inflammation	CCR2 blockade may prevent compensatory myeloid recruitment	(22)
PMN-MDSCs	CD11b, CD15, CD66b, LOX1, CXCR2, S100A8/A9, ARG1, MPO	IL-8/CXCR2, CysLTR1, G-CSF-like inflammation, lipid mediators	Suppress T-cell function through ARG1, ROS, metabolic stress, and inflammatory mediators	Neutrophil-rich and IL-8-high tumors often show poor ICB response	CXCR2 or CysLTR1 blockade may reduce granulocytic suppression	(19, 28)
Tumor-associated neutrophils	CXCR2, MPO, ELANE, S100A8/A9, LCN2, NET-related genes	IL-8/CXCR2, hypoxia, tumor inflammation, therapy-induced stress	Reduce tumor immunosensitivity; promote inflammatory remodeling and immune escape	Relevant in HCC and other neutrophil-rich tumors	TAN signatures may guide CXCR2-targeted or neutrophil-modulating therapy	(28, 181)
NLRP3-active MDSCs	NLRP3, IL1B, CASP1, ARG1, S100A8/A9	Microbial/fungal signals, inflammasome activation, IL-1β feedback	Enhance MDSC suppressive activity and promote tumor growth	Relevant in colorectal cancer and microbiome-associated inflammation	MDSC-targeted NLRP3 inhibition or IL-1β blockade may enhance anti-PD-1 therapy	(59, 60)
Bone marrow-trained suppressive myeloid precursors	IL4Rα, NRF2-associated genes, monocyte-lineage genes, oxidative stress programs	IL-4, tumor-derived systemic signals, oxidative stress, aging-related inflammation	Increase suppressive monocyte/TAM supply before tumor infiltration	Detectable in bone marrow or circulating myeloid precursors	Peripheral/bone marrow profiling may predict myeloid-dominant resistance	(14, 29, 30)
Senescence-associated myeloid suppressive niche	SASP cytokines, IL-6, CCL2, CXCLs, MDSC/TAM markers	Therapy-induced senescence, aging, cellular stress	Recruits and activates suppressive myeloid cells; promotes ICB resistance	May arise after chemotherapy, radiotherapy, or targeted therapy	Senolytic or senomorphic therapy may restore immune responsiveness	(78)

**Figure 4:**
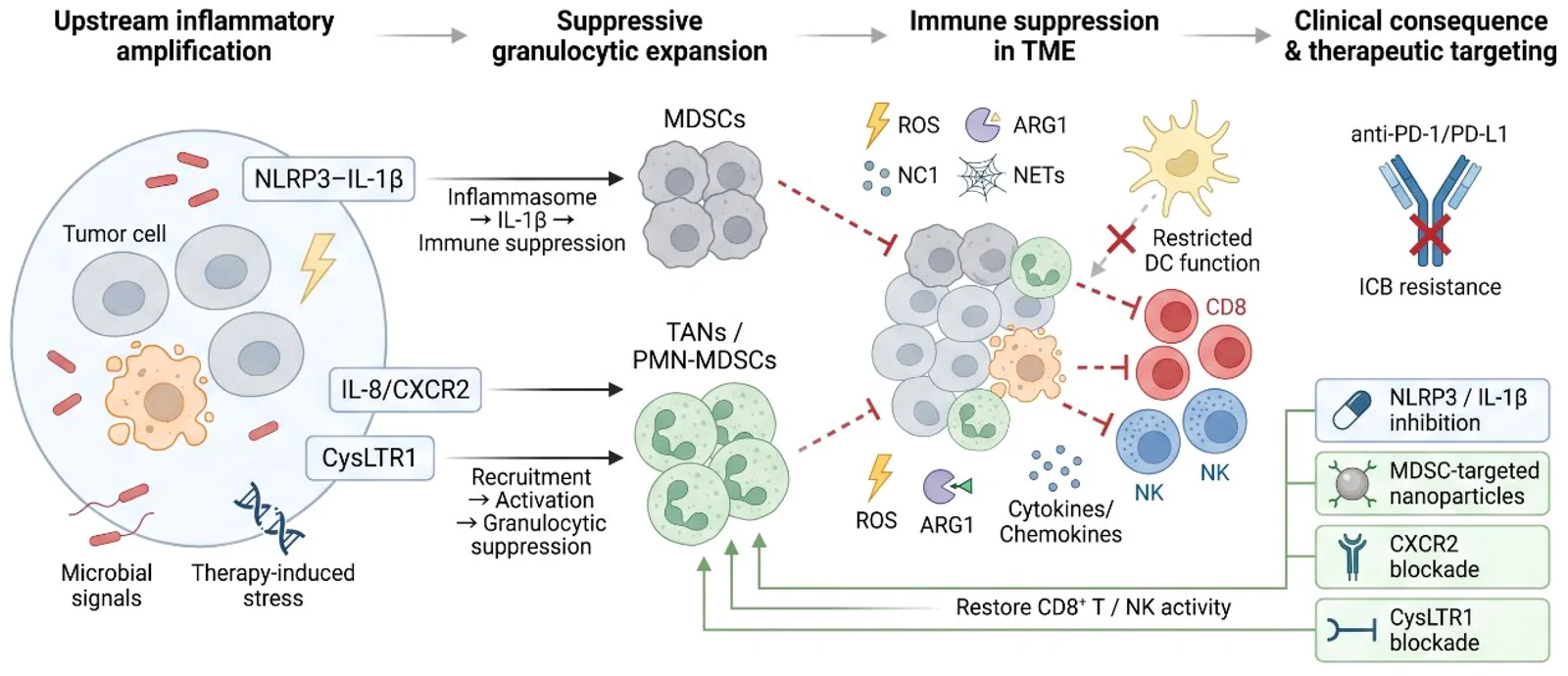
Suppressive granulocytic programs driven by inflammatory amplification. NLRP3–IL-1β, IL-8/CXCR2, and CysLTR1 signaling promote MDSC and TAN/PMN-MDSC expansion and activation. These cells suppress CD8^+^ T cells, NK cells, and dendritic-cell function through inflammatory mediators, ROS, ARG1, and NETs, contributing to ICB resistance and creating opportunities for myeloid-targeted intervention.

## 6 Stromal–myeloid inflammatory niches: CAF, macrophages, and spatial immune exclusion

Myeloid remodeling does not occur in isolation. Within tumors, suppressive myeloid cells are embedded in multicellular niches composed of cancer-associated fibroblasts, extracellular matrix, endothelial cells, malignant cells, hypoxic regions, metabolites, and lymphocytes (139, 189, 190). These stromal–myeloid niches are particularly important because they determine the spatial organization of antitumor immunity (191, 192). Even when CD8^+^ T cells are present, they may remain trapped in stromal regions, excluded from tumor nests, or positioned near suppressive macrophages and fibroblasts that limit their cytotoxic function. Therefore, immune resistance should be understood not only as a problem of immune-cell abundance, but also as a problem of spatial immune architecture. CAF–myeloid crosstalk is central to this architecture. CAFs can recruit monocytes and macrophages through chemokines, growth factors, extracellular matrix remodeling, and TGF-β-dependent signaling (192–195). In turn, TAMs can reinforce CAF activation by secreting inflammatory cytokines, osteopontin, complement components, growth factors, and matrix-remodeling mediators. This reciprocal communication stabilizes immunosuppressive niches in which stromal barriers, suppressive macrophages, and exhausted or excluded T cells coexist (139, 192, 196). These niches are often resistant to immune checkpoint blockade because T-cell reinvigoration alone does not dismantle the stromal and myeloid barriers that prevent effective tumor-cell killing.

### 6.1 CAF–macrophage crosstalk

CAF–macrophage crosstalk has emerged as a major determinant of immune checkpoint blockade response. In gastric cancer peritoneal metastases, CAF–macrophage interactions were shown to govern the response to immune checkpoint blockade (139). This study is highly relevant because peritoneal metastasis is characterized by dense stromal remodeling, macrophage accumulation, ascites-associated inflammation, and poor immunotherapy responsiveness. Rather than treating CAFs and TAMs as independent suppressive populations, this work demonstrates that their interaction creates a coordinated inflammatory niche that determines therapeutic outcome. The significance of this finding extends beyond gastric cancer. In many solid tumors, CAFs and macrophages form spatially organized networks that restrict T-cell infiltration and support tumor progression. CAFs may generate extracellular matrix barriers and chemokine gradients that trap T cells in stromal compartments, while TAMs suppress cytotoxic function through cytokines, checkpoint ligands, adenosine metabolism, and phagocytic or inflammatory programs (189, 197–201). When these two compartments interact, they create a self-reinforcing microenvironment in which stromal stiffness, macrophage suppression, and lymphocyte exclusion become mutually reinforcing.

TGF-β is one of the most important mediators of stromal–myeloid immune exclusion. In colorectal cancer, TGF-β was shown to build a dual immune barrier by impairing T-cell recruitment and instructing immunosuppressive SPP1^+^ macrophages (31). This provides a mechanistic explanation for why TGF-β-rich tumors often fail to respond to immune checkpoint blockade. TGF-β does not simply inhibit T-cell function directly. Instead, it alters the entire immune architecture by limiting lymphocyte entry while simultaneously shaping suppressive macrophage states (202–206). The result is a tumor microenvironment in which T cells are both numerically restricted and functionally opposed by myeloid cells. SPP1^+^ macrophages are particularly important in this stromal context. SPP1 is associated with extracellular matrix remodeling, fibroblast activation, angiogenesis, and immune suppression. When TGF-β induces or stabilizes SPP1^+^ TAM states, these macrophages can cooperate with CAFs to reinforce immune exclusion. This explains why SPP1^+^ TAM signatures often coincide with fibrotic, immune-excluded, or therapy-resistant tumors. Therapeutically, this suggests that targeting SPP1^+^ macrophages alone may be insufficient unless CAF-derived TGF-β signaling, extracellular matrix remodeling, and lymphocyte trafficking are also addressed. CAF–macrophage crosstalk therefore represents a multicellular mechanism of immune resistance. It converts inflammatory signaling into a spatially organized barrier that prevents cytotoxic lymphocytes from accessing and killing tumor cells. Biomarkers of this niche may include CAF abundance, TGF-β response signatures, SPP1^+^ TAM enrichment, complement-related macrophage programs, extracellular matrix density, and spatial proximity between CAFs and TAMs. These markers may be more informative than single immune-cell counts because they capture both cellular composition and tissue organization.

### 6.2 STAT3-dependent stromal inflammation

STAT3 is a central transcriptional mediator linking cytokine signaling, stromal inflammation, myeloid suppression, and therapy resistance. It can be activated downstream of IL-6 family cytokines, growth factors, oncogenic signaling, hypoxia-associated stress, and inflammatory feedback loops. In the tumor microenvironment, STAT3 activation can occur in malignant cells, CAFs, macrophages, MDSCs, endothelial cells, and other stromal compartments (118, 207–210). Because of this broad cellular distribution, STAT3 acts as a network-level regulator rather than a pathway limited to one cell type. In pancreatic ductal adenocarcinoma, combined MEK and STAT3 inhibition was shown to uncover stromal plasticity and overcome immunotherapy resistance (33). This study is important because PDAC is one of the most stromal-rich and immune-resistant cancers. Its dense fibroinflammatory microenvironment restricts T-cell infiltration, supports myeloid accumulation, and limits the efficacy of checkpoint blockade. By targeting MEK and STAT3 together, the study demonstrated that stromal inflammation and immune tolerance can be remodeled in a way that improves antitumor immunity. This finding has several implications. First, it shows that stromal inflammation is not fixed. CAF states and myeloid niches can be pharmacologically reprogrammed. Second, it highlights that single-pathway targeting may be insufficient because oncogenic MEK signaling and inflammatory STAT3 signaling can compensate for each other (208). Third, it suggests that effective immunotherapy combinations may need to target both tumor-intrinsic oncogenic pathways and microenvironmental inflammatory pathways. In PDAC and other desmoplastic tumors, this strategy may be necessary to convert an immune-excluded tumor into a more permissive immune ecosystem.

STAT3 also connects CAF biology with myeloid suppression. STAT3-dependent stromal inflammation can promote chemokine production, myeloid-cell recruitment, macrophage polarization, and MDSC accumulation. At the same time, myeloid cells can produce IL-6, IL-10, and other STAT3-activating cytokines that reinforce CAF activation and tumor-cell survival. This creates a feedback loop in which stromal and myeloid compartments maintain each other. Interrupting this loop may reduce immune suppression and increase the effectiveness of checkpoint blockade. From a biomarker perspective, STAT3 activation should be interpreted at the tissue-ecosystem level. Phosphorylated STAT3 in tumor cells, CAFs, or myeloid cells may have different functional meanings, but all can contribute to immune resistance. Therefore, spatial and single-cell analysis of STAT3 activity may help determine whether STAT3-targeted therapy is most likely to benefit patients with stromal-dominant, myeloid-dominant, or mixed inflammatory tumor microenvironments.

### 6.3 Treatment-induced inflammatory remodeling and adaptive myeloid feedback

In addition to pre-existing stromal–myeloid inflammation, anticancer therapy can generate a second layer of inflammatory remodeling. This treatment-induced remodeling differs from baseline inflammation because it arises after tumor-cell injury, tissue damage, immune activation, or therapy-induced cellular stress (22, 71, 132). Chemotherapy, radiotherapy, locoregional therapy, targeted therapy, immune checkpoint blockade, and innate immune agonists can initially promote antigen release, dendritic-cell activation, interferon production, and T-cell priming (211–214). However, these same interventions may also induce apoptotic debris, senescence-associated secretory programs, CCL2/CCL7- or CXCR2-related chemokines, and compensatory recruitment of TAMs, MDSCs, or TANs (22, 71, 215). Therefore, treatment-induced inflammation should be interpreted as an adaptive remodeling process rather than simply as enhanced antitumor immunity.

Therapy-induced senescence represents another mechanism of inflammatory niche formation. Senescent tumor or stromal cells produce a senescence-associated secretory phenotype that includes cytokines, chemokines, growth factors, matrix-remodeling enzymes, and inflammatory mediators. A 2024 study showed that senescence can drive immunotherapy resistance by inducing an immunosuppressive tumor microenvironment (78). Importantly, elimination of senescent cells improved immune homeostasis and restored sensitivity to immunotherapy. This suggests that therapy-induced senescence may be a hidden source of myeloid recruitment and immune suppression after anticancer treatment. STING activation provides a third example of therapy-induced inflammatory feedback. STING agonism is designed to activate innate antitumor immunity, particularly through type I interferon induction and dendritic-cell activation (216–220). However, in pancreatic cancer, activation of STING signaling by a gemcitabine-based nanocarrier also induced CCL2 and CCL7, which promoted CCR2-dependent recruitment of TAMs and MDSCs and contributed to immune resistance (66). Codelivery of a CCR2 antagonist helped overcome this negative feedback. This study clearly illustrates that even immune-stimulatory therapies can generate suppressive myeloid recruitment unless compensatory chemokine pathways are blocked.

These examples support a broader concept: therapy-induced inflammation is not automatically beneficial. The immune outcome depends on how tissue damage, cell death, cytokine release, and chemokine induction are interpreted by stromal and myeloid compartments. If therapy-induced inflammation promotes dendritic-cell activation and cytotoxic lymphocyte entry, it can enhance tumor immunity. If it preferentially recruits TAMs, MDSCs, TANs, or senescent stromal cells, it can generate adaptive resistance. Therefore, future combination therapies should be designed to pair tumor-damaging or innate immune-activating treatments with agents that prevent suppressive myeloid feedback. Therapy-induced inflammatory niches also provide opportunities for dynamic biomarkers. Measuring only pretreatment immune infiltration may miss important changes that occur after chemotherapy, radiation, embolization, senescence induction, or STING activation. Longitudinal analysis of TREM2^+^ TAMs, CCR2^+^ monocytes, MDSCs, TANs, senescence signatures, CCL2/CCL7 expression, IL-8/CXCR2 signaling, and spatial CD8^+^ T-cell exclusion may help identify when myeloid-targeted therapy should be added (221). In this way, treatment-induced inflammation can be transformed from a source of resistance into a rational guide for adaptive combination therapy.

Together, stromal–myeloid inflammatory niches explain why inflammation-rich tumors may still be immune resistant. CAFs, macrophages, MDSCs, TANs, senescent cells, and therapy-induced chemokine programs cooperate to shape the spatial and functional architecture of antitumor immunity. Targeting these niches requires more than T-cell reinvigoration; it requires dismantling the inflammatory and stromal circuits that continuously convert innate immune activation into myeloid-dominated suppression. **Figure 5** illustrates how stromal–myeloid inflammatory niches reshape the spatial architecture of antitumor immunity. It highlights CAF–TAM crosstalk, TGF-β/SPP1^+^ TAM signaling, STAT3-dependent stromal inflammation, and therapy-induced myeloid feedback as key mechanisms driving CD8^+^ T-cell exclusion and ICB resistance.

**Figure 5:**
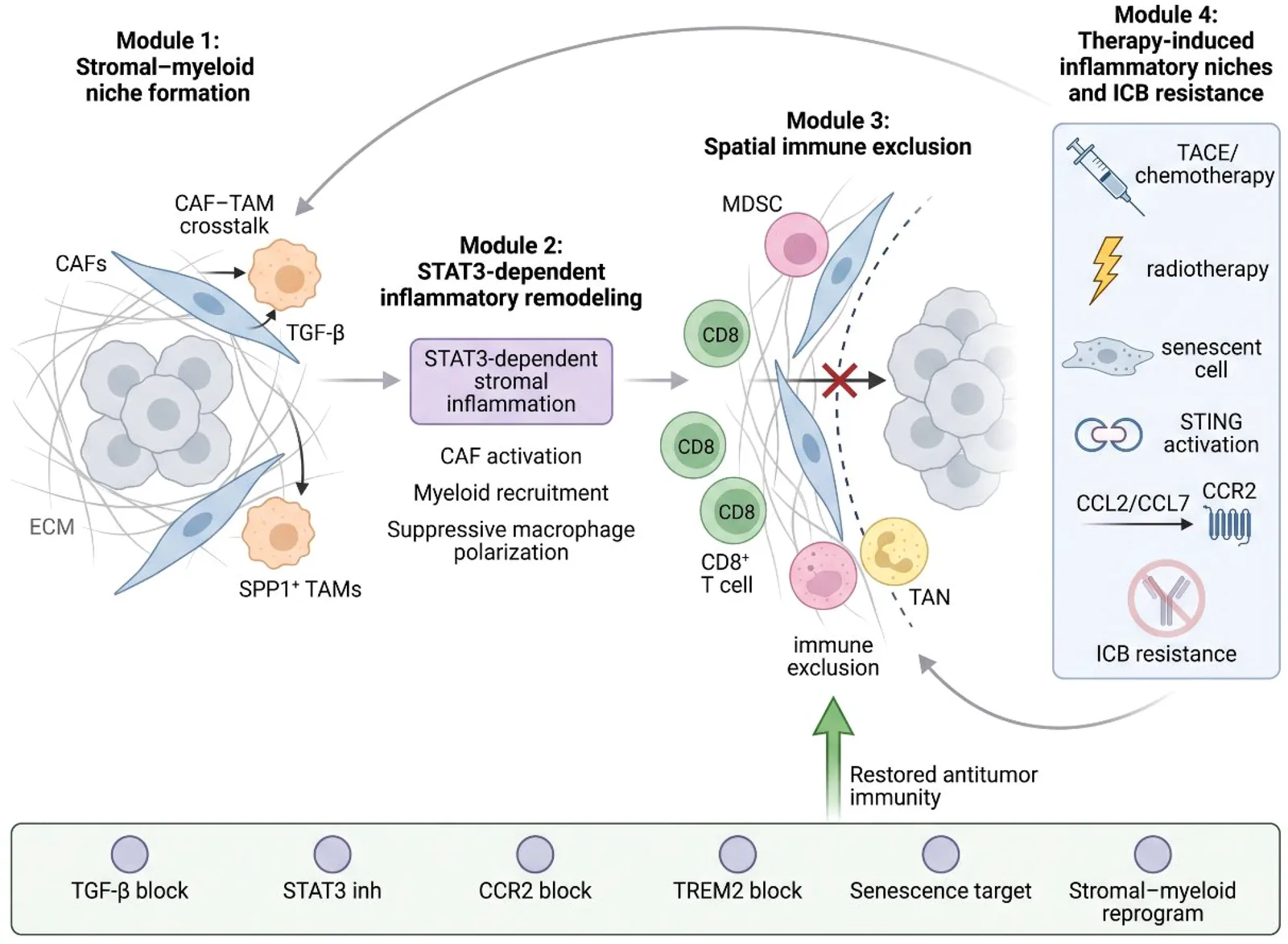
Stromal–myeloid inflammatory niches and spatial immune exclusion. CAF–TAM crosstalk, TGF-β/SPP1^+^ TAM signaling, STAT3-dependent stromal inflammation, and therapy-induced feedback remodel the tumor microenvironment. CAF/ECM barriers and suppressive myeloid cells restrict CD8^+^ T-cell infiltration, promote immune exclusion, and drive ICB resistance, supporting therapeutic strategies that reprogram stromal–myeloid circuits.

## 7 Myeloid remodeling as a mechanism of immunotherapy resistance

The mechanisms described above converge on several recurring resistance phenotypes. To avoid repeating pathway-specific details, this section focuses on how previously discussed programs, including NLRP3–IL-1β signaling, STING-associated CCR2^+^ myeloid recruitment, SPP1^+^ macrophage niches, and treatment-induced inflammatory remodeling, collectively generate resistance to immune checkpoint blockade (21, 22, 71, 222). These mechanisms are therefore discussed here as functional resistance outputs rather than as separate molecular pathways. Immune checkpoint blockade has transformed cancer therapy by restoring exhausted T-cell function and promoting durable antitumor immunity in selected patients (223–225). However, primary and acquired resistance remain common across many solid tumors. Although resistance is often attributed to tumor-intrinsic mechanisms, such as antigen-presentation defects, interferon pathway alterations, low tumor mutational burden, or impaired T-cell priming, increasing evidence indicates that myeloid remodeling is a major ecosystem-level mechanism of immunotherapy failure. In many tumors, checkpoint blockade does not fail because immune activation is absent. Rather, it fails because pre-existing or therapy-induced inflammatory signals are redirected toward suppressive myeloid compartments that limit T-cell infiltration, impair cytotoxic function, and reinforce immune exclusion. Myeloid-mediated resistance can be divided into two broad patterns. The first is baseline myeloid-dominant resistance, in which tumors are already enriched with suppressive macrophages, MDSCs, TANs, CAF-associated myeloid niches, or inflammatory chemokine programs before treatment begins. In this setting, immune checkpoint blockade is introduced into a tumor microenvironment that is structurally and functionally resistant to T-cell-mediated killing (192, 226, 227). The second is treatment-induced adaptive myeloid resistance, in which immune checkpoint blockade, chemotherapy, locoregional therapy, senescence, or innate immune activation induces compensatory inflammatory programs that recruit or activate suppressive myeloid cells. These two patterns are not mutually exclusive. A tumor with baseline myeloid inflammation may become even more resistant after therapy-induced tissue damage, cytokine release, or chemokine-driven myeloid recruitment (22, 71, 228).

### 7.1 Baseline myeloid-dominant resistance

Baseline myeloid-dominant resistance refers to a pretreatment immune state in which suppressive myeloid cells and stromal–myeloid inflammatory niches limit the efficacy of immune checkpoint blockade. In such tumors, CD8^+^ T cells may be numerically scarce, spatially excluded, or functionally restrained by macrophages, MDSCs, TANs, and CAFs. Importantly, these tumors may not be immunologically silent (229–233). They can contain abundant inflammatory cytokines, chemokines, and innate immune activation signatures. However, the dominant immune output is myeloid suppression rather than productive cytotoxic immunity. Gastric cancer peritoneal metastasis provides a clear example of baseline stromal–myeloid resistance. CAF–macrophage crosstalk in the tumor microenvironment has been shown to govern the response to immune checkpoint blockade in gastric cancer peritoneal metastases (139). This finding is important because it demonstrates that ICB responsiveness is not determined only by tumor-cell PD-L1 expression or T-cell abundance. Instead, the therapeutic outcome is shaped by a multicellular suppressive niche in which CAFs and macrophages communicate to reinforce immune exclusion and resistance. In this context, TAMs and CAFs should be viewed as cooperative components of a treatment-resistant ecosystem rather than independent suppressive cell types.

In hepatocellular carcinoma, IL-8/CXCR2 signaling represents another form of baseline myeloid resistance. IL-8 is a potent chemoattractant for neutrophil-lineage cells and immunosuppressive myeloid cells. Activation of the IL-8/CXCR2 pathway can promote MDSC trafficking and suppressive activity, thereby limiting the efficacy of immune checkpoint blockade. Targeting myeloid IL-8/CXCR2 signaling was shown to overcome immunotherapy resistance in HCC, supporting the view that chemokine-driven myeloid recruitment is a functional determinant of ICB failure rather than merely a correlative inflammatory feature (28). This mechanism is particularly relevant for tumors with elevated IL-8, neutrophil-rich infiltration, high CXCR2 activity, or PMN-MDSC/TAN enrichment. SPP1^hi macrophages provide another major example of baseline and evolving myeloid resistance. In advanced prostate cancer, myeloid-mediated immunotherapy resistance has been linked to the emergence of SPP1^hi tumor-associated macrophages (138). These macrophages are associated with immune suppression, adenosine signaling, and exhausted T-cell states. Their enrichment helps explain why metastatic castration-resistant prostate cancer is generally poorly responsive to checkpoint blockade despite the presence of immune infiltrates in some lesions. This study also illustrates that myeloid resistance is dynamic. As tumors progress, the macrophage compartment can evolve toward increasingly suppressive states that are not effectively reversed by T-cell checkpoint blockade alone. These examples show that baseline myeloid-dominant resistance is not a single mechanism. It can arise from CAF–macrophage crosstalk, IL-8/CXCR2-driven myeloid recruitment, SPP1^hi TAM-mediated immune suppression, TGF-β-associated immune exclusion, or combinations of these processes. However, they converge on a common outcome: effector lymphocytes are unable to access, persist in, or kill tumor cells effectively. Therefore, tumors with baseline myeloid-dominant resistance may require upfront combination strategies that pair immune checkpoint blockade with myeloid-targeted therapy, stromal remodeling, chemokine blockade, or macrophage reprogramming.

### 7.2 Treatment-induced adaptive myeloid resistance

Treatment-induced adaptive myeloid resistance occurs when therapy itself generates inflammatory conditions that favor suppressive myeloid recruitment or activation. This mechanism is especially important because many anticancer therapies are designed to increase immune activation. Checkpoint blockade, radiotherapy, chemotherapy, STING agonists, targeted therapy, and locoregional therapy can all induce tumor-cell death, antigen release, interferon signaling, tissue damage, and cytokine production. These effects may enhance antitumor immunity, but they can also create compensatory myeloid feedback loops that undermine therapeutic benefit. Interferon signaling provides a clear example of context-dependent adaptive resistance. Interferons are generally considered beneficial for antitumor immunity because they promote antigen presentation, dendritic-cell activation, and T-cell recruitment. However, in advanced renal cell carcinoma, myeloid cells were shown to mediate interferon-driven resistance to immunotherapy (47). This finding challenges the simple assumption that stronger interferon signaling always improves immunotherapy response. When interferon responses are sustained within a myeloid-dominated tumor ecosystem, they may reinforce suppressive myeloid programs and limit the effectiveness of checkpoint blockade. Thus, the functional meaning of interferon signatures depends on the cellular compartment in which they are expressed and the immune context in which they occur.

Tumor-associated neutrophils are also important mediators of adaptive immune resistance. In hepatocellular carcinoma, TANs were shown to attenuate tumor immunosensitivity (181). This suggests that neutrophil-lineage cells can actively limit the immune responsiveness of tumors, particularly in inflammatory liver cancer microenvironments. TANs may suppress T cells, alter metabolic conditions, remodel extracellular matrix, promote angiogenesis, and reinforce myeloid-dominant immune exclusion. Because TAN biology is highly context-dependent, future studies should distinguish antitumor neutrophil states from suppressive TAN/PMN-MDSC-like states and determine how therapy shifts this balance. Senescence-induced inflammation provides another mechanism of adaptive resistance. Cancer therapies can induce senescence in tumor cells and stromal cells. Although senescence may restrict tumor-cell proliferation, senescent cells produce a senescence-associated secretory phenotype that can recruit suppressive myeloid cells and reshape the tumor microenvironment. Senescence has been shown to drive immunotherapy resistance by inducing an immunosuppressive tumor microenvironment (78). Importantly, elimination of senescent cells restored immune homeostasis and improved response to immunotherapy. This finding indicates that therapy-induced senescence can convert treatment-induced stress into a persistent inflammatory niche that favors myeloid suppression.

STING activation further illustrates the paradox of therapy-induced innate immune activation. STING agonism is intended to promote type I interferon production, dendritic-cell activation, and T-cell priming. However, in pancreatic cancer, a STING-activating gemcitabine-based nanocarrier induced CCL2 and CCL7, resulting in CCR2-dependent recruitment of TAMs and MDSCs and subsequent immune resistance (66). Codelivery of a CCR2 antagonist helped overcome this compensatory myeloid recruitment. This study provides a clear therapeutic lesson: innate immune activation should be paired with strategies that block the suppressive myeloid feedback induced by that activation. Collectively, these findings show that adaptive myeloid resistance is an active response to therapy-induced inflammation. Tumor-cell killing, interferon induction, senescence, and innate immune activation can all improve antitumor immunity under some conditions, but they can also recruit or activate TAMs, MDSCs, and TANs. Therefore, the success of combination therapy depends not only on stimulating immunity, but also on controlling the myeloid consequences of that stimulation. A rational treatment strategy should ask not only “Does this therapy activate immunity?” but also “Which myeloid populations does this therapy recruit, expand, or reprogram?”.

## 8 Biomarkers of innate immune signaling–driven myeloid remodeling

Biomarkers of myeloid remodeling are needed to identify patients who are unlikely to benefit from immune checkpoint blockade alone and to guide rational combination therapy. Traditional immune biomarkers, such as PD-L1 expression, tumor mutational burden, microsatellite instability, and CD8^+^ T-cell infiltration, provide useful but incomplete information (79, 234, 235). They do not fully capture the suppressive myeloid circuits, stromal organization, inflammatory cytokine states, or systemic myelopoietic programs that determine whether antitumor immunity can be executed. Therefore, biomarkers of innate immune signaling–driven myeloid remodeling should include tissue markers, spatial markers, peripheral blood markers, systemic inflammatory markers, and dynamic treatment-induced changes. A useful biomarker framework should answer four questions. First, which suppressive myeloid populations are present? Second, which inflammatory pathways are driving their recruitment or activation? Third, where are these myeloid cells located relative to CD8^+^ T cells, tumor cells, CAFs, and blood vessels? Fourth, do these biomarkers change after therapy in a way that predicts adaptive resistance? Addressing these questions requires integration of bulk transcriptomics, single-cell RNA sequencing, spatial transcriptomics, multiplex immunofluorescence, flow cytometry, circulating cytokine analysis, and longitudinal sampling.

### 8.1 Representative tissue and spatial biomarkers

Tissue biomarkers can identify suppressive myeloid states within tumor lesions. For tumor-associated macrophages, candidate markers include TREM2, SPP1, C1QC, NLRP3, IL1B, C3AR1, CD163, MRC1, and macrophage-associated complement genes. TREM2 marks a suppressive macrophage state that has been linked to reduced NK-cell activity in lung cancer, myeloid reprogramming in glioblastoma, and impaired CD8^+^ T-cell infiltration after TACE in HCC (125). SPP1 identifies a macrophage program associated with stromal remodeling, immune exclusion, adenosine signaling, and ICB resistance, as shown in prostate cancer and stromal–myeloid tumor niches (31, 138, 139). NLRP3 and IL1B identify inflammasome-active macrophage states that may promote IL-1β-driven inflammatory amplification and tumor progression (57, 58).

For MDSCs and TANs, useful tissue markers may include CXCR2, IL8, S100A8, S100A9, ARG1, MPO, LOX1, LCN2, CXCL1, CXCL2, and neutrophil elastase-related programs. The IL-8/CXCR2 axis is particularly important because it links inflammatory chemokine signaling to myeloid recruitment and immunotherapy resistance in HCC (28). TAN-related markers may be especially informative in tumors with neutrophil-rich inflammation, such as subsets of HCC, where TANs can reduce tumor immunosensitivity (181). However, marker interpretation requires caution because neutrophil-lineage cells are heterogeneous, and not all TANs are functionally suppressive. Therefore, neutrophil markers should ideally be combined with functional signatures, such as ARG1, ROS-associated programs, NET-related genes, or T-cell exclusion features.

Inflammatory pathway biomarkers can help identify upstream drivers of myeloid remodeling. These may include IL-1β, IL-4Rα, STAT3 activation, TGF-β response signatures, interferon response signatures, NLRP3 inflammasome components, CCL2/CCR2 signaling, IL-8/CXCR2 signaling, and CysLTR1-related lipid inflammatory pathways. For example, IL-4Rα expression or IL-4-responsive myeloid programs may indicate systemic type 2 inflammatory myelopoiesis (29). TGF-β response signatures may identify tumors with stromal immune exclusion and SPP1^+^ macrophage instruction (31). Interferon signatures should be interpreted carefully because they may reflect either productive antitumor immunity or myeloid-mediated adaptive resistance depending on cellular localization and treatment context (47).

Spatial biomarkers are particularly important because myeloid suppression is often organized anatomically. A tumor may contain CD8^+^ T cells but still be resistant if those cells are confined to stromal regions, excluded from malignant epithelial nests, or positioned near suppressive TAMs and CAFs. Relevant spatial indicators include the distance between SPP1^+^ TAMs and CD8^+^ T cells, proximity between CAFs and TAMs, localization of TREM2^+^ macrophages near treatment-induced debris, concentration of TANs at invasive margins, and whether CD8^+^ T cells are located in the tumor parenchyma or retained within stromal compartments. CAF–macrophage proximity may be especially relevant in gastric cancer peritoneal metastasis, where stromal–myeloid crosstalk governs ICB response (139).

Because individual markers can be nonspecific, composite biomarker panels are likely to be more useful than single proteins. For example, a myeloid-resistance panel could combine SPP1^+^ TAM abundance, TGF-β response, CAF density, CD8^+^ T-cell exclusion, and IL-8/CXCR2 activity. Another panel could combine NLRP3, IL1B, MDSC markers, and circulating IL-1β to identify inflammasome-driven myeloid suppression. A third panel could integrate TREM2^+^ TAMs, therapy-induced debris, and CD8^+^ T-cell exclusion to identify patients who may benefit from TREM2-targeted or macrophage-reprogramming strategies.

### 8.2 Peripheral and systemic biomarkers: risk assessment rather than treatment selection

Peripheral biomarkers are needed because myeloid remodeling begins not only inside tumors but also in the bone marrow and circulation. Blood-based markers may provide an accessible window into systemic myelopoiesis, inflammatory cytokine states, and treatment-induced myeloid adaptation. Commonly used clinical indicators include neutrophil-to-lymphocyte ratio, monocyte counts, platelet-to-lymphocyte ratio, circulating MDSC frequency, and serum inflammatory cytokines (236). Although these markers are not highly specific, they may reflect broad shifts toward systemic inflammation and myeloid dominance. More mechanistic systemic biomarkers should focus on circulating monocyte subsets, neutrophil-lineage cells, MDSCs, and myeloid progenitor-like populations. IL-4-driven bone marrow myelopoiesis suggests that IL-4Rα expression on myeloid progenitors or monocyte-lineage cells may indicate systemic type 2 inflammatory training (29). IL-1α-driven emergency myelopoiesis in aging suggests that IL-1-related cytokines, myeloid progenitor expansion, and age-associated inflammatory signatures may identify patients with a host environment predisposed to myeloid-dominant tumor progression (30). Tumor-induced progenitor dysregulation and NRF2-associated programs further suggest that chromatin accessibility, oxidative stress signatures, or transcriptional states in circulating or bone marrow myeloid precursors could function as early biomarkers of suppressive macrophage output (14).

CysLTR1-related myelopoiesis also highlights the potential value of lipid-inflammatory biomarkers. If leukotriene signaling regulates tumor-promoting myeloid output, then circulating lipid mediators, CysLTR1 expression, or downstream inflammatory gene signatures may help identify patients who could benefit from CysLTR1-targeted therapy or other interventions aimed at suppressive granulocytic programs (19). Similarly, serum IL-8 may serve as a clinically accessible indicator of CXCR2-dependent neutrophil or PMN-MDSC recruitment, particularly in HCC and other tumors where IL-8/CXCR2 signaling contributes to ICB resistance (28). Dynamic biomarkers may be even more informative than baseline measurements. Therapy can induce interferon responses, senescence-associated inflammatory programs, STING-dependent chemokines, TREM2^+^ macrophage accumulation, TAN expansion, and MDSC recruitment. Therefore, blood and tissue should ideally be sampled before treatment, early during therapy, and at progression. For example, rising CCL2/CCL7 after STING activation may indicate compensatory CCR2^+^ myeloid recruitment and the need for CCR2 blockade (66). Increased senescence-associated cytokines after chemotherapy or targeted therapy may suggest senescence-driven myeloid suppression and potential benefit from senolytic or senomorphic strategies (78). Expansion of circulating neutrophils or PMN-MDSC-like cells during treatment may indicate emerging granulocytic resistance.

Ultimately, biomarkers of innate immune signaling–driven myeloid remodeling should be interpreted as integrated immune-ecosystem readouts rather than isolated laboratory values. A high neutrophil-to-lymphocyte ratio, elevated IL-8, increased circulating MDSCs, SPP1^+^ TAM enrichment, CAF–TAM proximity, and stromal CD8^+^ T-cell exclusion may together identify a tumor that is unlikely to respond to immune checkpoint blockade alone. Conversely, patients with low suppressive myeloid burden, intratumoral CD8^+^ T-cell localization, and limited inflammatory feedback may be more likely to respond to T-cell-directed therapy without intensive myeloid targeting. In this way, biomarker-guided patient stratification can help shift the field from empiric immunotherapy combinations toward rational, mechanism-based myeloid reprogramming strategies.

## 9 Therapeutic targeting: from pathway inhibition to myeloid reprogramming

Therapeutic targeting of innate immune signaling–driven myeloid remodeling should not be understood as simple suppression of inflammation. In cancer, innate immune pathways can generate both antitumor immunity and tumor-promoting myeloid suppression. Therefore, the therapeutic goal is not to eliminate innate immune activation globally, but to redirect it away from chronic inflammatory amplification and toward productive antitumor immunity (237–241). This requires a shift from pathway-centered treatment to ecosystem-centered myeloid reprogramming. A useful therapeutic framework can be organized around four principles. First, upstream inflammatory drivers that initiate suppressive myelopoiesis or myeloid recruitment should be blocked when they dominantly promote immune suppression. Second, inflammasome and IL-1β-dependent amplification loops should be interrupted in tumors where NLRP3-active TAMs or MDSCs sustain inflammatory resistance. Third, suppressive macrophage, neutrophil, and MDSC states should be reprogrammed rather than simply depleted, because myeloid cells can also support antigen presentation, tissue remodeling, and immune activation when properly redirected. Fourth, myeloid-targeted interventions should be combined with immune checkpoint blockade in a biomarker-guided manner, particularly in tumors with baseline myeloid dominance or treatment-induced adaptive myeloid resistance. However, the clinical translation of myeloid-targeted therapy has been uneven, and several strategies that were promising in preclinical models have shown limited efficacy, context-dependent benefit, or clinically important toxicity in patients. Therefore, therapeutic opportunities should be discussed together with their translational limitations.

### 9.1 Blocking upstream inflammatory drivers

One therapeutic strategy is to block the upstream inflammatory signals that initiate suppressive myeloid remodeling. These signals include IL-4/IL-4Rα, IL-1α/IL-1β, TGF-β, interferon-related adaptive resistance programs, and lipid-inflammatory pathways such as CysLTR1 signaling. These pathways act before or during myeloid-cell differentiation and can therefore influence the entire downstream immune landscape. The IL-4/IL-4Rα axis is a representative example. IL-4 has traditionally been associated with type 2 immunity and alternative macrophage activation, but recent evidence indicates that it can also act at the level of bone marrow myelopoiesis. IL-4 signaling through IL-4Rα can drive pro-tumorigenic myeloid output, generating monocyte-lineage cells that later support immunosuppressive tumor microenvironments (29). Blocking IL-4Rα therefore has the potential to intervene early in the myeloid remodeling process. Rather than only reprogramming mature TAMs after they enter the tumor, IL-4Rα blockade may reduce the systemic production of suppressive myeloid cells and enhance the effectiveness of immune checkpoint blockade.

IL-1 signaling is another attractive upstream target. IL-1α and IL-1β can promote emergency myelopoiesis, recruit suppressive myeloid cells, and amplify inflammatory feedback. In aging-associated cancer progression, IL-1α-driven emergency myelopoiesis has been shown to promote tumor growth by increasing the accumulation of myeloid progenitor-like cells in tumors (30). This suggests that IL-1 blockade may be particularly relevant in older patients, tumors with high inflammatory myelopoiesis, or settings where therapy-induced tissue damage increases IL-1 signaling. Targeting IL-1 may reduce the systemic generation and local accumulation of suppressive myeloid populations, especially when combined with immunotherapy.

TGF-β represents a more complex but highly important therapeutic target. In colorectal cancer, TGF-β was shown to create a dual immune barrier by impairing T-cell recruitment and instructing immunosuppressive SPP1^+^ macrophages (31). This dual activity provides a strong rationale for combining TGF-β inhibition with immune checkpoint blockade in immune-excluded tumors. However, TGF-β has broad roles in tissue homeostasis, fibrosis, immune tolerance, and epithelial biology. Therefore, therapeutic targeting must be carefully timed and guided by biomarkers such as stromal TGF-β response signatures, SPP1^+^ TAM enrichment, CAF density, and spatial exclusion of CD8^+^ T cells.

Interferon-related adaptive resistance requires a different therapeutic logic. Interferon signaling is essential for antigen presentation and antitumor immunity, but persistent interferon responses in myeloid cells can also mediate immunotherapy resistance. In advanced renal cell carcinoma, myeloid cells were shown to drive interferon-associated resistance to immune checkpoint blockade (47). This does not support indiscriminate interferon inhibition. Instead, it suggests that therapeutic strategies should distinguish productive interferon signaling in dendritic cells and tumor-reactive lymphocytes from suppressive interferon programs in myeloid cells. Future approaches may need to combine checkpoint blockade with selective targeting of interferon-induced myeloid resistance pathways rather than global interferon suppression.

CysLTR1 signaling provides an additional upstream target linking inflammatory lipid mediators to tumor-promoting myelopoiesis. Targeting cysteinyl leukotriene receptor 1 was shown to reprogram tumor-promoting myelopoiesis and overcome immune checkpoint therapy resistance (19). This finding is important because it expands therapeutic attention beyond canonical cytokines. Lipid inflammatory pathways may shape neutrophil-lineage and monocyte-lineage output, influence PMN-MDSC accumulation, and regulate the inflammatory tone of the tumor-bearing host. CysLTR1 blockade may therefore be useful in tumors where leukotriene-associated inflammation contributes to suppressive myeloid generation and ICB resistance.

Together, upstream inflammatory blockade aims to prevent the formation of a suppressive myeloid supply chain. This strategy may be particularly valuable in patients with systemic inflammatory signatures, emergency myelopoiesis, high circulating monocytes or neutrophils, elevated IL-1 or IL-8, strong TGF-β activity, or evidence of tumor-driven bone marrow reprogramming.

### 9.2 Targeting inflammasome and IL-1β-dependent myeloid suppression

A second strategy is to target inflammasome-dependent inflammatory amplification, especially the NLRP3–caspase-1–IL-1β axis. This pathway is particularly relevant when tumor-associated macrophages or MDSCs convert danger signals, apoptotic debris, microbial products, or therapy-induced damage into chronic inflammatory suppression. In these settings, inflammasome inhibition may reduce tumor-promoting inflammation while improving the efficacy of T-cell-directed immunotherapy. Efferocytosis provides an important example of inflammasome-driven tumor promotion. Although clearance of apoptotic cells is usually associated with tissue homeostasis and inflammation resolution, in tumors efferocytosis can activate myeloid NLRP3 inflammasome signaling and IL-1β secretion, thereby promoting tumor growth (57). This finding suggests that tumor-cell death alone is not sufficient to generate effective antitumor immunity. The immune consequence of cell death depends on whether dying tumor cells are processed by dendritic cells in an immunogenic manner or by suppressive macrophages that activate NLRP3-dependent inflammatory amplification. NLRP3^+^ macrophages may also represent a clinically relevant suppressive state. In head and neck squamous cell carcinoma, NLRP3 expression in tumor-associated macrophages was associated with poor prognosis and tumor-promoting activity (58). This supports the use of NLRP3, IL1B, caspase-1-related markers, and macrophage-associated inflammasome signatures as biomarkers for selecting patients who may benefit from inflammasome-targeted therapy. Importantly, NLRP3 inhibition should be considered in the context of cellular localization. NLRP3 activity in macrophages, MDSCs, epithelial cells, or tumor cells may have distinct effects, and therapeutic benefit may depend on whether the dominant inflammasome-active compartment is myeloid.

MDSC-directed inflammasome targeting is especially promising. MDSC-targeting gold nanoparticles were shown to enhance anti-PD-1 tumor immunotherapy by inhibiting NLRP3 inflammasomes (60). This approach is conceptually attractive because it avoids broad systemic immunosuppression and instead delivers inflammasome inhibition to a suppressive myeloid population. By reducing NLRP3 activity in MDSCs, such strategies may weaken IL-1β-dependent suppression, improve CD8^+^ T-cell activity, and increase sensitivity to checkpoint blockade. This illustrates the broader value of targeted delivery platforms for myeloid reprogramming. Microbiome-associated inflammation can also converge on this pathway. In colorectal cancer, Candida tropicalis was shown to enhance MDSC immunosuppressive function through IL-1β-dependent mechanisms and thereby promote tumor progression (59). This finding suggests that inflammasome-targeted therapy may be particularly relevant in tumors influenced by microbial dysbiosis, barrier disruption, fungal colonization, or chronic mucosal inflammation. It also raises the possibility that microbiome modulation, antifungal strategies, IL-1β blockade, and MDSC-targeted therapy could be combined in selected gastrointestinal cancers. Therapeutically, the NLRP3–IL-1β axis can be targeted at several levels, including NLRP3 activation, caspase-1 activity, IL-1β maturation, IL-1 receptor signaling, and myeloid-cell-specific delivery of inhibitors. The challenge is to suppress chronic tumor-promoting inflammasome activity without impairing beneficial innate immune activation needed for antigen presentation and immune priming. Therefore, inflammasome-targeted therapy should ideally be guided by biomarkers such as NLRP3^+^ TAMs, IL1B expression, MDSC infiltration, serum IL-1β, microbial dysbiosis, and poor response to PD-1/PD-L1 blockade.

### 9.3 Reprogramming suppressive TAMs and TANs

A third therapeutic strategy is to reprogram suppressive myeloid states after they have already formed within the tumor microenvironment. This approach is particularly important because many tumors are diagnosed after suppressive TAM, MDSC, and TAN niches are already established. Rather than simply depleting all myeloid cells, reprogramming strategies aim to convert suppressive myeloid cells into inflammatory, antigen-presenting, phagocytic, or immune-supportive states. TREM2 is one of the most promising macrophage reprogramming targets. In glioblastoma, TREM2 inhibition was shown to trigger antitumor activity of myeloid cells and improve tumor control (131). This finding is important because glioblastoma is a myeloid-dominated tumor in which conventional immune checkpoint blockade has limited efficacy. TREM2 targeting may shift macrophages away from suppressive lipid-handling and tissue-repair programs toward more inflammatory and antitumor states. Similarly, in hepatocellular carcinoma, TREM2^+^ TAMs were shown to suppress CD8^+^ T-cell infiltration after transarterial chemoembolization, and TREM2 deficiency enhanced anti-PD-L1 efficacy (132). These findings suggest that TREM2 blockade may be particularly useful in tumors with therapy-induced debris, macrophage accumulation, and poor CD8^+^ T-cell infiltration.

SPP1-associated macrophage programs provide another major therapeutic opportunity. In prostate cancer, SPP1^hi TAMs were linked to the evolution of myeloid-mediated immunotherapy resistance and adenosine-associated immune suppression (138). Targeting the SPP1-related axis may therefore require more than neutralizing osteopontin itself. It may involve disrupting macrophage–stromal interactions, adenosine metabolism, extracellular matrix remodeling, or TGF-β-driven macrophage instruction. Because SPP1^+^ TAMs often exist within CAF-rich and immune-excluded niches, SPP1-related targeting may be most effective when combined with stromal remodeling or TGF-β pathway inhibition. For neutrophil-lineage suppression, IL-8/CXCR2 is a central target. In HCC, targeting myeloid IL-8/CXCR2 signaling was shown to overcome immunotherapy resistance (28). This strategy aims to reduce recruitment and suppressive activity of CXCR2^+^ myeloid cells, including TANs and PMN-MDSC-like cells. Because IL-8 is clinically measurable and often associated with poor immunotherapy response, the IL-8/CXCR2 axis may provide both a therapeutic target and a biomarker-guided patient-selection strategy. CCR2 blockade is another important myeloid-recruitment strategy, particularly for monocyte-derived macrophages and MDSCs. In pancreatic cancer, STING activation induced CCL2 and CCL7 expression, which promoted CCR2-dependent recruitment of TAMs and MDSCs and contributed to immune resistance (66). Codelivery of a CCR2 antagonist with a STING-activating nanocarrier helped block this compensatory myeloid recruitment. This provides a clear therapeutic principle: when innate immune activation induces chemokine-mediated myeloid feedback, the recruitment pathway should be blocked at the same time as immune activation is induced.

STAT3 is another therapeutic node connecting stromal inflammation, myeloid suppression, and tumor-intrinsic signaling. In pancreatic ductal adenocarcinoma, combined MEK and STAT3 inhibition remodeled stromal plasticity and overcame immunotherapy resistance (33). This finding suggests that effective myeloid reprogramming may require targeting both tumor-cell oncogenic pathways and inflammatory transcriptional programs within stromal and myeloid compartments. STAT3 inhibition may reduce suppressive cytokine feedback, macrophage polarization, MDSC accumulation, and CAF-mediated immune exclusion. CysLTR1 also belongs in the category of myeloid reprogramming targets because its blockade can reshape tumor-promoting myelopoiesis and improve response to immune checkpoint therapy (19). Unlike targets that act only within mature tumor-infiltrating myeloid cells, CysLTR1 may influence both systemic myeloid output and local inflammatory myeloid function. This dual activity makes it particularly interesting for tumors with granulocytic inflammation, leukotriene signaling, and poor ICB responsiveness.

Overall, TAM and TAN reprogramming strategies should be chosen according to the dominant suppressive myeloid state. TREM2-targeted approaches may be appropriate for lipid-rich, debris-rich, macrophage-dominated tumors. SPP1-related strategies may be useful in stromal, fibrotic, immune-excluded tumors. CXCR2 blockade may be preferable in neutrophil-rich tumors. CCR2 blockade may be needed when monocyte recruitment is driven by CCL2/CCL7. STAT3 inhibition may be valuable when stromal inflammation and myeloid suppression are tightly linked. CysLTR1 blockade may be relevant when lipid-inflammatory signaling drives tumor-promoting myelopoiesis.

### 9.4 Combination strategies with immune checkpoint blockade

The most clinically relevant application of myeloid-targeted therapy is combination with immune checkpoint blockade. Because myeloid remodeling can mediate both primary and adaptive resistance to PD-1/PD-L1 or CTLA-4 blockade, rational combinations should be designed according to the specific type of myeloid barrier present in each tumor. Broadly, these combinations can be divided into three categories: ICB plus inflammatory pathway blockade, ICB plus myeloid recruitment blockade, and ICB plus myeloid-state reprogramming.

The first category is ICB plus inflammatory pathway blockade. This approach is suitable when upstream inflammatory drivers maintain suppressive myeloid programs. Examples include blockade of IL-1 signaling in tumors with IL-1α/IL-1β-driven emergency myelopoiesis, TGF-β inhibition in immune-excluded tumors with SPP1^+^ TAM instruction, STAT3 inhibition in stromal-inflammatory tumors, and CysLTR1 targeting in tumors with leukotriene-driven tumor-promoting myelopoiesis (242). The goal is to reduce the inflammatory signals that continuously generate or stabilize suppressive myeloid populations, thereby allowing checkpoint blockade to restore T-cell function more effectively.

The second category is ICB plus myeloid recruitment blockade. This approach is appropriate when therapy-induced or tumor-intrinsic chemokines recruit suppressive monocytes, macrophages, MDSCs, or neutrophils. CCR2 blockade can be used to prevent CCL2/CCL7-driven recruitment of TAMs and MDSCs, particularly in settings where STING activation or chemotherapy induces compensatory monocyte recruitment (66). CXCR2 blockade can be used to reduce TAN and PMN-MDSC accumulation in IL-8-high tumors, including HCC models of immunotherapy resistance (28). These strategies are especially relevant for tumors in which immune activation paradoxically increases suppressive myeloid infiltration.

The third category is ICB plus myeloid-state reprogramming. This approach does not primarily block recruitment, but instead changes the functional state of myeloid cells already present in tumors. TREM2 inhibition can reprogram suppressive macrophages toward more antitumor activity, as shown in glioblastoma and HCC contexts (131, 132). NLRP3 inhibition in MDSCs can reduce inflammasome-dependent immune suppression and enhance anti-PD-1 therapy (60). SPP1-related targeting may weaken stromal–macrophage immune exclusion and adenosine-associated resistance. These approaches are particularly relevant for established tumors with dense TAM or MDSC infiltration.

Timing is a major unresolved issue in myeloid-targeted combination therapy. In tumors with strong baseline myeloid-dominant resistance, myeloid-targeted therapy may need to be administered before or together with ICB to open the immune microenvironment. In tumors where myeloid resistance emerges after treatment, dynamic monitoring may identify the optimal time to add CCR2, CXCR2, NLRP3, TREM2, or senescence-targeted interventions. For example, STING activation may require concurrent CCR2 blockade to prevent CCL2/CCL7-driven suppressive recruitment, whereas therapy-induced senescence may require delayed senolytic or senomorphic intervention after tumor-cell killing has occurred. Patient selection is equally important. A tumor with high SPP1^+^ TAMs, CAF density, and CD8^+^ T-cell exclusion may benefit from TGF-β or stromal–myeloid targeting. A tumor with elevated IL-8, CXCR2^+^ neutrophils, and PMN-MDSC signatures may benefit from CXCR2 blockade (180). A tumor with high NLRP3, IL1B, and MDSC infiltration may benefit from inflammasome-targeted therapy. A tumor with therapy-induced CCL2/CCL7 induction may benefit from CCR2 blockade. A tumor with TREM2^+^ TAM accumulation after locoregional therapy may benefit from macrophage reprogramming. Therefore, myeloid-targeted immunotherapy should be biomarker-guided rather than empirically added to all patients.

In summary, therapeutic targeting of innate immune signaling–driven myeloid remodeling requires a shift from immune activation alone to immune ecosystem correction. Successful treatment will likely require combining checkpoint blockade with interventions that block upstream inflammatory drivers, prevent suppressive myeloid recruitment, inhibit inflammasome amplification, and reprogram established TAM, MDSC, and TAN states. The ultimate goal is not simply to reduce myeloid cells, but to convert the tumor microenvironment from a chronic inflammatory suppressive niche into a spatially permissive and functionally productive immune ecosystem. Because myeloid remodeling can arise from distinct inflammatory drivers, recruitment pathways, stromal niches, and suppressive cell states, therapeutic strategies should be matched to the dominant resistance mechanism in each tumor context. **Table 3** summarizes potential myeloid-targeted strategies and their rationale for combination with immune checkpoint blockade.

**Table 3:** Therapeutic strategies for targeting innate immune signaling–driven myeloid remodeling.

Therapeutic logic	Target/pathway	Candidate strategy	Expected immune effect	Best-fit biomarker or tumor context	Combination rationale with ICB	Representative PMID
Block systemic type 2 inflammatory myelopoiesis	IL-4/IL-4Rα	IL-4Rα blockade; dupilumab-like strategy	Reduces immunosuppressive monocyte output and increases CD8^+^ T-cell infiltration	IL-4/IL-4Rα-high tumors; systemic monocyte expansion; type 2 inflammatory TME	Prevents suppressive myeloid supply while ICB restores T-cell function	(29)
Suppress aging-associated emergency myelopoiesis	IL-1α/IL-1R	IL-1 pathway blockade	Reduces age-associated myeloid progenitor expansion and tumor-promoting inflammation	Older patients; IL-1-high tumors; emergency myelopoiesis signatures	May reduce baseline myeloid resistance before or during ICB	(30)
Interrupt TGF-β-driven stromal–myeloid exclusion	TGF-β pathway	TGF-β inhibition; stromal-targeted combinations	Improves T-cell recruitment and reduces SPP1^+^ suppressive macrophage instruction	TGF-β-high, CAF-rich, immune-excluded tumors	Opens spatial barriers and improves cytotoxic T-cell access after ICB	(31)
Modulate IFN-related adaptive resistance	Myeloid IFN-response programs	Selective targeting of myeloid IFN-resistance pathways	Prevents persistent IFN signaling from reinforcing suppressive myeloid states	RCC or tumors with myeloid-localized IFN resistance signatures	Preserves beneficial antigen presentation while reducing adaptive resistance	(47)
Block lipid-inflammatory myelopoiesis	CysLTR1	CysLTR1 antagonist; leukotriene-pathway targeting	Reprograms tumor-promoting myelopoiesis and reduces granulocytic suppression	CysLTR1-high tumors; neutrophil/PMN-MDSC-rich ICB-resistant tumors	May convert ICB-resistant tumors into ICB-sensitive tumors	(19)
Inhibit macrophage inflammasome amplification	NLRP3/caspase-1/IL-1β	NLRP3 inhibitor; caspase-1 inhibitor; IL-1β blockade	Reduces IL-1β-driven inflammatory amplification and tumor-promoting TAM activity	NLRP3^+^ TAMs; IL1B-high tumors; debris-rich tumors	Prevents therapy-induced cell death from feeding suppressive inflammation	(57, 58)
Target inflammasome-active MDSCs	NLRP3 in MDSCs	MDSC-targeted nanoparticles; myeloid-directed NLRP3 inhibition	Weakens MDSC-mediated T-cell suppression and improves anti-PD-1 efficacy	MDSC-high, NLRP3/IL1B-high tumors	Directly disables suppressive myeloid cells while ICB reinvigorates T cells	(60)
Target microbiome-driven MDSC inflammation	Candida–IL-1β–MDSC axis	Microbiome modulation; antifungal strategy; IL-1β blockade	Reduces microbial/fungal enhancement of MDSC suppressive function	CRC with fungal dysbiosis or mucosal inflammation	May improve ICB or inflammatory-pathway combinations in GI tumors	(59)
Reprogram TREM2^+^ macrophages	TREM2	Anti-TREM2 antibody; TREM2 inhibition	Shifts TAMs toward antitumor activity; restores NK/CD8^+^ T-cell function	TREM2^+^ TAM-high tumors; GBM; lung cancer; post-TACE HCC	Reduces macrophage-mediated suppression that limits ICB efficacy	(125, 131, 132)
Target SPP1^+^ stromal macrophage programs	SPP1-associated TAM axis; adenosine-related pathways	SPP1-axis targeting; adenosine A2A pathway inhibition; stromal–myeloid combinations	Reduces stromal immune exclusion, T-cell exhaustion, and myeloid-mediated ICB resistance	SPP1^+^ TAM-high, CAF-rich, immune-excluded tumors	Weakens TAM–CAF suppressive niches while ICB restores T-cell activity	(138, 139)
Block neutrophil/PMN-MDSC recruitment	IL-8/CXCR2	CXCR2 antagonist; IL-8 pathway blockade	Reduces TAN/PMN-MDSC recruitment and granulocytic immune suppression	IL-8-high, CXCR2^+^, TAN/PMN-MDSC-rich tumors; HCC	Prevents granulocytic suppression that limits ICB response	(28, 181)
Prevent monocyte/TAM/MDSC feedback after STING activation	CCL2/CCL7–CCR2 axis	CCR2 antagonist combined with STING agonist or STING-activating nanocarrier	Blocks compensatory TAM/MDSC recruitment after innate immune activation	STING-treated tumors with induced CCL2/CCL7; PDAC	Allows STING-induced immune activation without suppressive myeloid feedback	(22)
Reprogram stromal inflammation	MEK/STAT3	Combined MEK and STAT3 inhibition	Remodels CAF plasticity and stromal–myeloid inflammatory tolerance	PDAC; desmoplastic, STAT3-active, immune-excluded tumors	Converts stromal-resistant tumors into more ICB-responsive ecosystems	(33)
Eliminate or modulate senescence-driven suppressive niches	Senescence/SASP pathways	Senolytic or senomorphic therapy	Reduces SASP-driven myeloid recruitment and restores immune homeostasis	Therapy-induced senescence; SASP-high tumors; adaptive ICB resistance	Prevents treatment-induced inflammatory niches from blocking ICB response	(78)
Biomarker-guided myeloid reprogramming	Integrated myeloid signatures	Match therapy to dominant myeloid program	Converts myeloid-dominant tumors into immune-permissive ecosystems	TREM2^+^ TAM, SPP1^+^ TAM, NLRP3^+^ MDSC, CXCR2^+^ TAN, CAF–TAM proximity	Enables rational rather than empirical ICB combinations	Multiple

### 9.5 Clinical limitations and safety concerns of myeloid-targeted therapy

Although myeloid-targeted strategies are strongly supported by preclinical studies, their clinical translation has been challenging. One major limitation is that pharmacodynamic depletion or modulation of myeloid cells does not necessarily translate into durable tumor regression (243–245). For example, CSF1/CSF1R blockade can reduce macrophage survival or alter TAM composition, but several clinical studies in advanced solid tumors have shown limited antitumor activity despite pharmacodynamic evidence of target engagement. The anti-CSF1R antibody AMG 820 showed pharmacodynamic activity but limited antitumor efficacy as monotherapy, and the combination of AMG 820 with pembrolizumab was not sufficiently active for further evaluation in selected advanced solid tumors (245). Similarly, pexidartinib combined with durvalumab showed limited antitumor activity in advanced colorectal and pancreatic cancer, possibly because off-target effects on FLT3-dependent dendritic-cell differentiation may counteract immune activation. These findings indicate that simple TAM depletion or CSF1R blockade may be insufficient, and in some contexts may impair immune-supportive myeloid or dendritic-cell compartments (245, 246).

Safety is another important translational barrier. Pexidartinib provides a clear example: although it has clinical activity in tenosynovial giant cell tumor, the FDA review identified serious hepatotoxicity concerns, including cases of irreversible liver injury, and recommended a boxed warning and restricted distribution under a REMS program (247–249). This illustrates that targeting macrophage-lineage biology can affect tissue-resident macrophage functions and normal organ homeostasis, particularly in the liver. Therefore, myeloid-targeted agents should not be viewed as immunologically neutral add-ons to checkpoint blockade. Chemokine-axis blockade also illustrates the gap between preclinical rationale and clinical benefit. CCR2 inhibition with PF-04136309 combined with nab-paclitaxel and gemcitabine in metastatic pancreatic ductal adenocarcinoma raised concerns for synergistic pulmonary toxicity and did not show an efficacy signal beyond chemotherapy (250). Similarly, the CXCR2 antagonist navarixin plus pembrolizumab in advanced solid tumors showed limited efficacy and was closed at a prespecified interim analysis for lack of efficacy, highlighting the difficulty of translating neutrophil/MDSC recruitment blockade into broad clinical benefit. These examples suggest that future myeloid-targeted combinations should be developed more cautiously (245, 246, 250, 251). Key requirements include biomarker-defined patient selection, confirmation that the intended suppressive myeloid population is actually dominant in the tumor, longitudinal pharmacodynamic monitoring, careful dose and schedule optimization, and early assessment of organ-specific toxicity. Rather than broadly depleting myeloid cells, future strategies should aim to selectively reprogram pathological myeloid states while preserving dendritic-cell function, antimicrobial defense, tissue repair, and homeostatic macrophage activity.

## 10 Conclusions and future perspectives

This review supports four major conclusions. First, innate immune signaling in cancer should be interpreted according to context, duration, cellular compartment, and treatment status rather than as uniformly antitumor or protumor. Acute and spatially restricted innate activation may support antigen presentation and effector-cell recruitment, whereas chronic, systemic, stromal, or therapy-induced inflammation can redirect myeloid cells toward suppressive and immune-resistant programs. Second, cancer-associated myeloid remodeling should be viewed as a systemic-to-local process. Tumor- and host-derived inflammatory cues can influence myeloid development, circulating myeloid output, tumor infiltration, and spatial organization within stromal niches. This perspective helps explain why tumors with similar T-cell biomarkers may differ substantially in their response to immune checkpoint blockade (233). Third, biomarker and therapeutic development should move beyond broad marker enumeration or empirical immune stimulation. More informative strategies should focus on mechanism-based myeloid programs, spatial immune organization, treatment-induced inflammatory feedback, and longitudinal changes during therapy. Similarly, therapeutic approaches should aim to reprogram pathological myeloid states while preserving beneficial innate immune functions, including antigen presentation, host defense, tissue repair, and organ homeostasis. Several unresolved questions remain. It is still unclear which inflammatory signals are dominant drivers rather than bystander features in specific tumor types, how pre-existing inflammation differs from treatment-induced remodeling over time, which patients require upfront myeloid-targeted combinations, and which patients may benefit from adaptive or sequential intervention. Future studies should integrate single-cell profiling, spatial analysis, peripheral immune monitoring, functional validation, and carefully designed clinical trials to define when, where, and how innate immune signaling–driven myeloid remodeling should be therapeutically targeted.
